# Chiral Perylene Bisimide Dyes by Interlocked Arene Substituents in the Bay Area

**DOI:** 10.1002/chem.202101877

**Published:** 2021-07-07

**Authors:** Rebecca Renner, Bernhard Mahlmeister, Olga Anhalt, Matthias Stolte, Frank Würthner

**Affiliations:** ^1^ Institut für Organische Chemie Universität Würzburg Am Hubland 97074 Würzburg Germany; ^2^ Center for Nanosystems Chemistry (CNC) Universität Würzburg Theodor-Boveri-Weg 97074 Würzburg Germany

**Keywords:** chirality, circular polarized luminescence, perylene bisimide dyes, Suzuki coupling

## Abstract

A series of perylene bisimide (PBI) dyes bearing various aryl substituents in 1,6,7,12 bay positions has been synthesized by Suzuki cross‐coupling reaction. These molecules exhibit an exceptionally large and conformationally fixed twist angle of the PBI *π‐*core due to the high steric congestion imparted by the aryl substituents in bay positions. Single crystal X‐ray analyses of phenyl‐, naphthyl‐ and pyrenyl‐functionalized PBIs reveal interlocked *π‐π*‐stacking motifs, leading to conformational chirality and the possibility for the isolation of enantiopure atropoisomers by semipreparative HPLC. The interlocked arrangement endows these molecules with substantial racemization barriers of about 120 kJ mol^−1^ for the tetraphenyl‐ and tetra‐2‐naphthyl‐substituted derivatives, which is among the highest racemization barriers for axially chiral PBIs. Variable temperature NMR studies reveal the presence of a multitude of up to fourteen conformational isomers in solution that are interconverted via smaller activation barriers of about 65 kJ mol^−1^. The redox and optical properties of these core‐twisted PBIs have been characterized by cyclic voltammetry, UV/Vis/NIR and fluorescence spectroscopy and their respective atropo‐enantiomers were further characterized by circular dichroism (CD) and circular polarized luminescence (CPL) spectroscopy.

## Graphical abstract

**The interlocked***π‐π*‐stacking motif of bay‐tetraarylated perylene bisimides endows these molecules with chirality and rich conformational isomerism. These structural features are elucidated by X‐ray crystallography and NMR. Optical spectroscopy including CD and CPL provide insights into their functional properties.

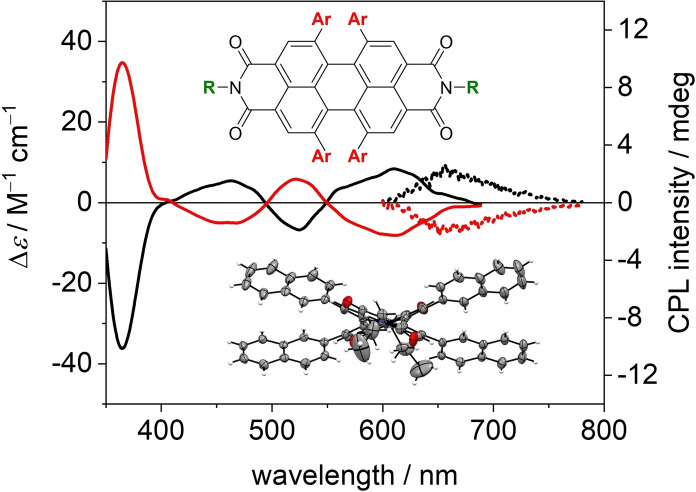

## Introduction

Perylene bisimides (PBIs) have been known for more than a century.^[1]^ Initially used as vat dyes and pigments with superb tinctorial strength and photostability,[^2^, ^3^, ^4^] more applications have emerged over the last decades due to the unique properties of these molecules. These include outstanding fluorescence quantum yields,^[5]^ excellent stability against environmental influences and high electron affinity, allowing for fairly stable radical anionic states[^6^, ^7^] as required for ambient stable n‐type semiconductors.[^8^, ^9^, ^10^] Their easy accessibility and the tailored properties arising by core functionalization promoted the application of PBI dyes in organic light emitting diodes (OLEDs),[^11^, ^12^] organic thin‐film transistors (OTFTs),[^10^, ^13^] and organic lithium batteries.[^14^, ^15^] In particular, a wide range of applications of PBIs were recently explored in organic solar cells (OSCs) where they found use in interlayers,^[16]^ as sensitizers,^[17]^ or as non‐fullerene acceptors (NFAs).[^18^, ^19^] Furthermore, PBIs are applied as fluorescence labels for bioimaging[^20^, ^21^] and as fluorescence dyes for single‐molecule spectroscopy and microscopy.^[22]^

The molecular properties of PBIs can be tailored by substitution in the bay^[2]^ or headland (ortho)[^23^, ^24^] positions whilst the imide residues have only a minor influence on the molecular properties and are commonly used to control the solubility and guide the molecular arrangement in supramolecular aggregates or in the solid state.^[25]^ Most significantly, the substitution in the central bay positions not only changes the molecular structure along with the optical and electronic properties but influences solubility and molecular packing as well. Usually, the corresponding bis‐ and tetra‐bay‐halogenated PBIs[^26^, ^27^] are used as precursors for the bay functionalization. By nucleophilic substitution of the halogen atoms with alcohol‐, amino‐, and thiol‐groups, a broader variety of PBI chromophores with adjustable optical and redox properties are accessible.[^2^, ^4^, ^28^] Another option for the variation of functional groups in bay position of a halogenated PBI are palladium‐catalyzed C−C coupling reactions like the Suzuki coupling[^27^, ^29^, ^30^] and the Sonogashira reaction.[^31^, ^32^] However, due to the steric vicinity of the substituents in 1 and 12 as well as in 6 and 7 position, respectively, the synthesis of tetra‐bay‐substituted PBIs via coupling reactions remains challenging.^[27]^ Depending on the steric demand of each substituent, a twist of the two naphthalene subunits of the PBI *π*‐scaffold is observed, resulting in axial chirality. Variation of the twist angle affects both solubility and electronic properties of the molecule. The angle between the two naphthalimide sub‐planes can be as large as 37.2° for the tetrabromo substituted PBI, as proven by single crystal X‐ray crystallography.^[8]^ Despite the close proximity and steric demand of the four bay substituents, fast interconversion between the *P*‐ and *M*‐atropoenantiomers is typically observed for tetrasubstituted PBIs, hampering the separation of the two isomers.^[33]^ Strategies hitherto applied to increase the racemization barrier comprise the fixation of one atropoenantiomer with aryloxy‐substituents into a macrocyclic structure[^34^, ^35^, ^36^] or the introduction of groups with large van‐der‐Waals radii like bromine or phenyl.^[33]^ The isolation of the respective *P*‐ and *M*‐atropoenantiomers at ambient conditions requires a racemization barrier higher than 93 kJ mol^−1^.^[33]^ A systematic experimental investigation of the racemization process has previously been conducted for halogenated PBIs but to the best of our knowledge, no similar reports are found for tetraarylated PBIs, which have first been synthesized by Zhu and coworkers in 2006 via Suzuki‐cross coupling reaction^[27]^ and were further explored by Hoffmann and co‐workers.[^29^, ^30^]

Inspired by the intriguing crowding of four aryl groups in bay area and in this regard surprisingly high yields for the synthesis of 1,6,7,12‐tetraphenyl‐PBIs by fourfold Suzuki coupling,[^27^, ^29^, ^30^] we herein systematically investigate the influence of a broader variety of substituents attached at the four bay positions on the properties of these chromophores, i.e. their racemization barriers, UV/Vis/NIR absorption and fluorescence as well as circular dichroism (CD) and circular polarized luminescence (CPL). Furthermore, we present the first single crystal X‐ray analyses of different tetraarylated PBIs that reveal a *π‐π*‐stacking enforced interlocked motif and twist angles of up to 36.6°. Our systematic investigations by temperature‐dependent NMR spectroscopy provide insights into the activation energies for racemization and reveal an amazing variety of conformational isomers that are stabilized by *π‐π*‐stacking interactions.

## Results and Discussion

### Synthesis

**Scheme 1:**
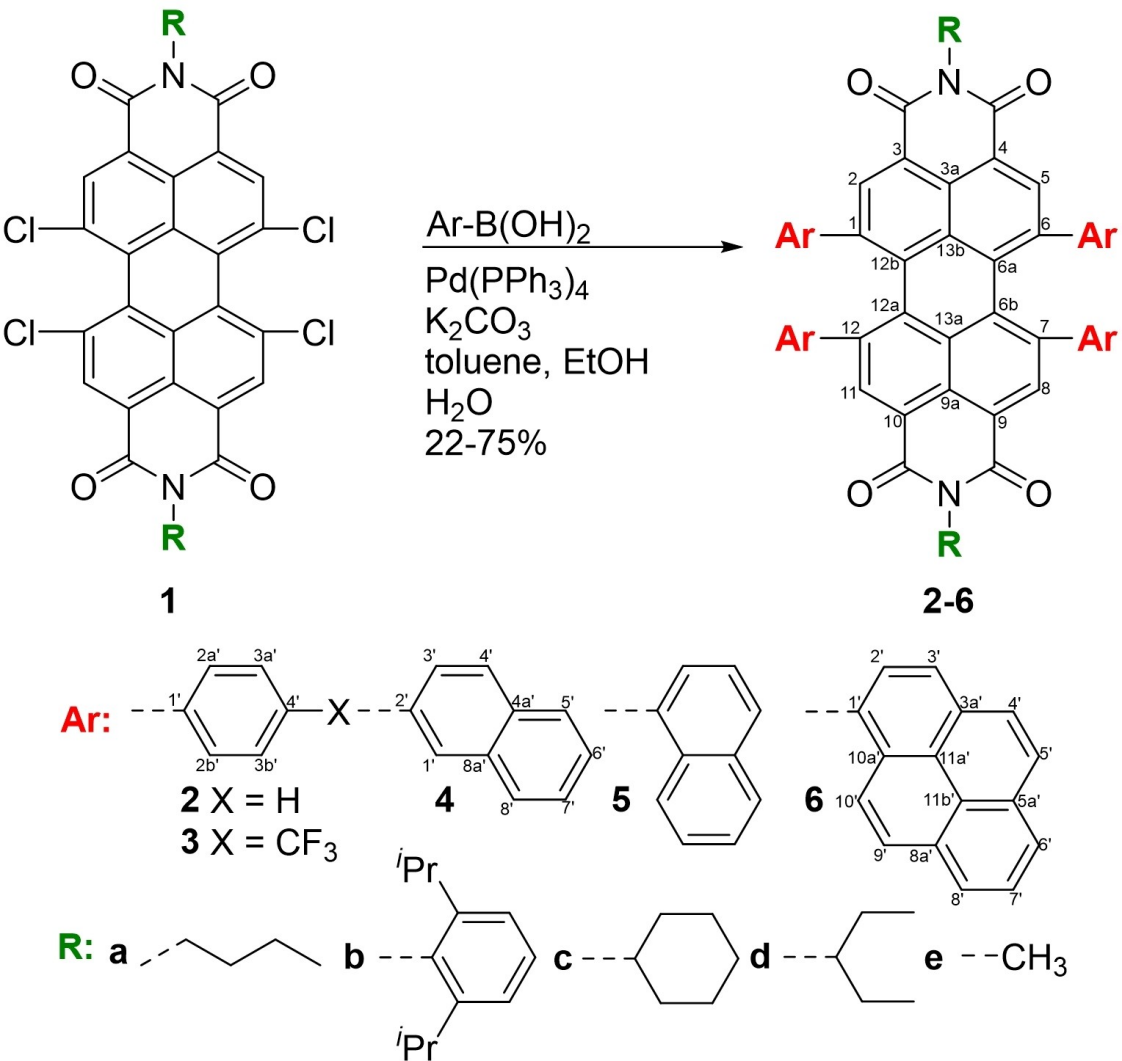
Synthesis of a series of tetraarylated (Ar, red) PBIs **2 a**–**2 e**, **3 a**, **4 a**, **5 a** and **6 a** by Suzuki cross‐coupling bearing different imide substituents (R, green) with labelling of the carbon atoms of the chromophore core.

The whole series of tetraarylated PBIs investigated in this study was synthesized by Suzuki‐coupling of the respective 1,6,7,12‐tetrachloro PBI with the corresponding arylboronic acid. The reactions were conducted in the presence of the catalyst Pd(PPh_3_)_4_ and potassium carbonate in a solvent mixture of toluene, ethanol and water by application of three freeze‐pump‐thaw cycles, to ensure deoxygenated conditions (Scheme 1). Utilizing this straightforward synthetic approach, a variety of bay‐tetraarylated PBIs are synthesized. These include literature‐reported derivatives **2 a**–**c** and new derivatives with different substituents (**2 d**–**e**, **3 a**, **4 a**, **5 a**, **6 a**). PBIs **2 a**, **2 b** and **2 c**, which were previously reported in literature,[^29^, ^30^, ^37^] could be resynthesized with increased yields for all three derivatives of 73 %, 75 % and 66 %, respectively. The other two derivatives with phenyl substituents in bay‐position, **2 d** and **2 e**, could be obtained in slightly lower yields of 46 % and 51 %, respectively, which might arise from lower conversion from the starting material to the product and losses during column chromatography and HPLC purification due to the lower solubility. Molecule **3 a** with the electron‐withdrawing trifluoromethyl group attached in the phenyl‐*para*‐position could be obtained in a similarly high yield of 60 %. Upon extension of the aryl residues, the yield decreases notably. Derivative **4 a** with 2‐naphthyl substituents was obtained in a yield of 28 %, whereas for **5 a** with 1‐naphthyl substituents 38 % of the product could be isolated. The largest 1‐pyrene substituent in **6 a** gives the lowest yield of all synthesized derivatives of 22 %. These observations can be rationalized by the investigation of the side products formed in this reaction, that reveal increasing amounts of triarylated PBI, due to the dehalogenation of the fourth bay‐position. Additionally, a mixture of the different double arylated and double dehalogenated PBIs can be isolated after the workup of the reaction mixture. This dehalogenation competes with the arylation reaction and becomes favored upon increasing steric demand of the aryl substituents as seen within the series **2 a** (73 %), **4 a** (28 %) to **6 a** (22 %).[^27^, ^30^]

### Structural analysis in the solid state

**Figure 1:**
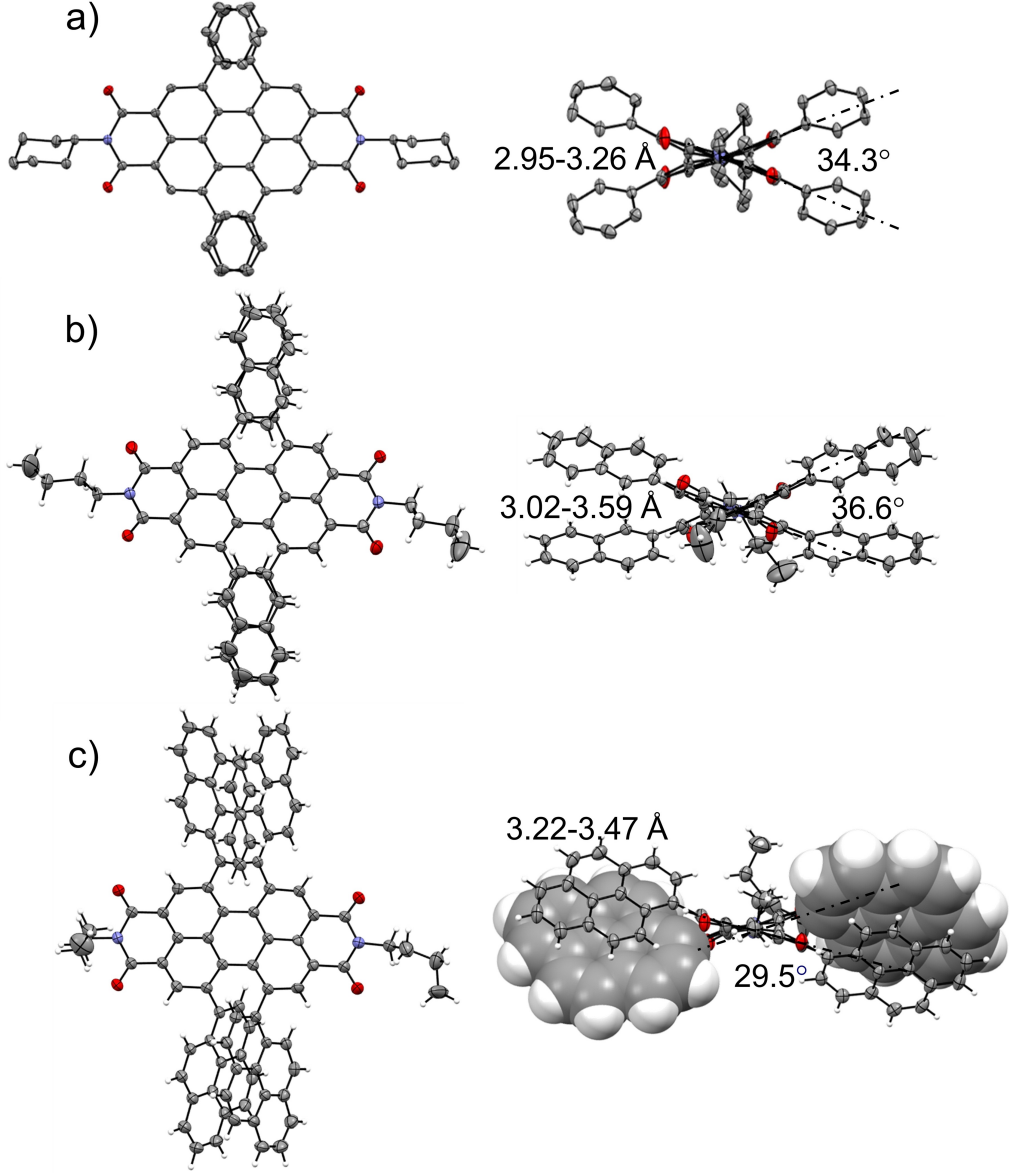
Molecular structures of each *M*‐atropoenantiomer of the single‐crystal structures of a) **2 c**, b) **4 a** and c) **6 a** in top view onto the PBI‐*π*‐system (left) and along the *N*,*N*’‐axis (right). The ellipsoids are set to 50 % probability (C: gray, O: red, N: blue, H: white). Disorder of the imide residues as well as solvent molecules are omitted for clarity.^[41]^

For the characterization in the solid state, single crystals of the tetraarylated PBIs **2 c**, **4 a** and **6 a** suitable for X‐ray structure analysis could be obtained. This was realized by slow evaporation of either a chloroform (**2 c**) or chlorobenzene (**4 a**, **6 a**) solution of the racemic mixture of the respective PBI that was carefully covered by a layer of *n*‐hexane. The molecular structures in the crystal provide unambiguous evidence for the heavily twisted PBI *π*‐scaffolds as well as the *π*‐*π*‐stacking enforced interlocked arrangement of the aryl substituents in bay position. While **2 c** crystallizes in the monoclinic C2/c space group, **4 a** and **6 a** crystallize in the triclinic P‐1 space group. All crystals include both atropoenantiomers (*M* and *P*) and the structures of **2 c** and **4 a** contain additional solvent molecules in the unit cell. For comparison, only the *M*‐atropoenantiomers for each derivative are depicted in Figure 1 while omitting the small disorder of the imide substituents as well as all solvent molecules. In **4 a** and **6 a**, only the conformational isomers with the highest symmetry with respect to the orientation of the four bay substituents are found in the respective single crystal structure. This might originate from their predominance in solution compared to the other possible isomers as observed by ^1^H NMR spectroscopy (see below) and their higher propensity to crystallize, in the most symmetric structures I for **4 a** (Figure S5) and **6 a** (Figure S6), respectively. This conformational selection might be guided additionally by the maximization of *π‐π*‐interactions between the two *π*‐stacked aryl residues in each of the bay areas.

The distortion of the PBI cores can be assessed by the consideration of the naphthalimide units as two planes and was measured to be 34.3°, 36.6° and 29.5° for **2 c**, **4 a** and **6 a**, respectively (Table S1). These values are among the highest reported twist angles for PBI chromophores to be observed in single crystal structures. The twist angle of 36.6° of **4 a** is only slightly smaller than the record value of 37.2° for the tetrabromo‐substituted PBI,^[8]^ but significantly larger than the structurally related tetraarylated peropyrenes reported by Chalifoux, which exhibit a twist angle of 29°.^[38]^ This emphasizes the high strain in the PBI core imposed by the attachment of four directly conjugated aryl substituents. While the bromine atoms in the tetrabromo‐substituted congener exhibit a very large steric crowding directly in bay position due to the large van‐der‐Waals radius of bromine, the aryl substituents are able to establish intramolecular *π‐π*‐stacking interactions between 2.95 to 3.59 Å as shown in Figure 1. A second method to assess the distortion of the perylene core is to measure the dihedral angle of the four carbon atoms in the bay area ∠(C1, C12b, C12a and C12) (numbering according to Scheme 1). The dihedral angles for **2 c** and **6 a** differ slightly for both sides of the PBI, indicating a broken symmetry in the solid state, presumably due to packing effects. In contrast in **4 a** both bay areas exhibit the same dihedral angle of 33.5° due to the presence of three perpendicular *C*_2_ axes resulting in overall *D*_2_ symmetry of the core. In **2 c** the dihedral angles were found to be 32.7° and 33.4° respectively, while the difference is less pronounced in **6 a** with 29.2° and 29.9° with the larger pyrene substituents. Such broken symmetry has been reported before for tetraphenoxy‐PBIs[^38^, ^39^] where it is an intrinsic feature of the perylene core rather than a packing effect in the solid state.^[40]^ The carbon‐carbon bond distances within the perylene core in all three molecules are comparable to previously reported PBIs. The larger carbon‐carbon distances between the two naphthalene subunits compared to the shorter bonds within the naphthalene subunits explain the higher susceptibility of the PBI scaffold to distortions in the bay area.^[38]^

**Figure 2:**
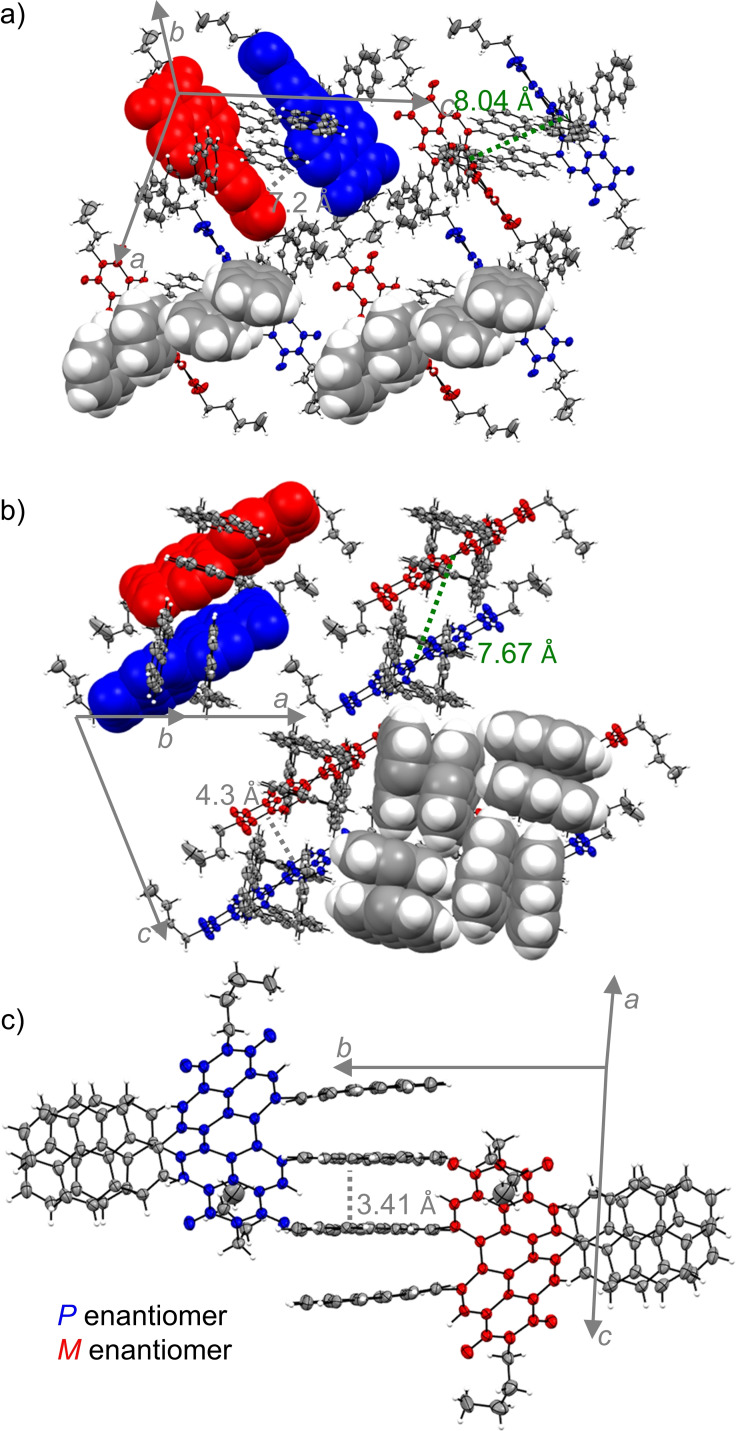
a) Molecular packing of **4 a**, as well as b) side view onto the PBI *π*‐surface and c) side view onto the *π*‐stacked pyrene substituents of **6 a** in their respective single crystal structures. PBI atropoenantiomers are colored in blue (*P*) and red (*M*). The ellipsoids are set to 50 % probability (C: gray, O: red, N: blue, H: white). Disorder of the imide residues as well as solvent molecules are omitted for clarity.^[41]^

While the single crystal X‐ray structure of PBI **2 c** contains a large amount of solvent molecules and shows almost no interactions between the individual chromophores, the molecular packing in the crystal of **4 a** shows a slip‐stacked packing arrangement of *P*‐ and *M*‐PBI atropoenantiomers at a center‐to‐center distance (*r*_C‐C_) of 8.04 Å with chlorobenzene occupying the solvent accessible voids within the unit cell (Figure 2a). The most interesting crystal structure is, however, the one of tetra‐pyrenyl‐substituted PBI **6 a** that contains only a small void with only one disordered chlorobenzene molecule and is accordingly guided to a large degree by intermolecular interactions among the chromophores and their bay aryl‐substituents. Similar as for **4 a**, also for **6 a** the characteristic slip‐stacked packing arrangement of *P*‐ and *M*‐PBI chromophores at *r*_C‐C_ of 7.67 Å can be observed, that is here, however, directed by additional CH‐*π*‐interactions between the pyrene bay substituents (Figure 2b). It is interesting to note that the co‐facially stacked pyrene dimers are organized in tetrameric squares in these crystals, obviously directed by CH‐*π* interactions and that this kind of packing motif of *π*‐stacked dimers interconnected by CH‐*π*‐interactions mimic the molecular arrangement found in single crystals of pristine pyrene.^[42]^ Furthermore, along another crystallographic axis a quadruple *π*‐stacking motif consisting of two pyrene dimers can be identified (Figure 2c). Thus, it can be concluded, that the crystal packing in **6 a** is strongly influenced by the pyrene substituents rather than by the PBI core, while in **2 c** and **4 a** the core governs the crystal packing more than the respective aryl substituents.

### Conformational analysis in solution

The characterization of the different bay‐substituted PBIs by ^1^H NMR spectroscopy in 1,1,2,2‐tetrachloroethane‐*d* ^2^ and CD_2_Cl_2_ revealed structural differences in solution in dependence of the type and connection of the respective bay substituents. It is important to state, that the subsequently discussed ^1^H NMR spectra were always obtained, regardless of the fraction of isolated material after purification or its temperature treatment. At room temperature, the signals assigned to the *ortho*‐ and *meta*‐position of the phenyl substituents in **2 a** are broadened (7.08 ppm and 6.70 ppm), while the signal of the *para*‐proton (7.22 ppm) and the protons in *ortho*‐position of the PBI core (8.24 ppm) exhibit sharp signals. Upon heating, the broad *ortho*‐ and *meta*‐proton signals become a sharp doublet and triplet respectively, indicating a fast exchange between the protons, while upon cooling two sets of signals can be observed, which are assigned to slow exchange of the protons due to decelerated rotation of the phenyl units (Figure S1).^[43]^ The coalescence temperature *T*_c_ for the observed process is 303 K and the rotational barrier Δ*G* ^≠^ of 64.7 kJ mol^−1^ is comparable to values obtained for the rotation of different imide substituents in PBIs around the terminal C−N single bond, indicating the feasibility of this rotation at room temperature.^[44]^ DFT calculations (Figure S4) provide a reasonable approximation for the rotational barrier and insights into the distortions required for the rotation of the aryl groups and the energetically more demanding enantiomerization that resembles the related processes in carbohelicenes^[45]^ and in tetramethoxy perylene bisimides.^[46]^

**Figure 3:**
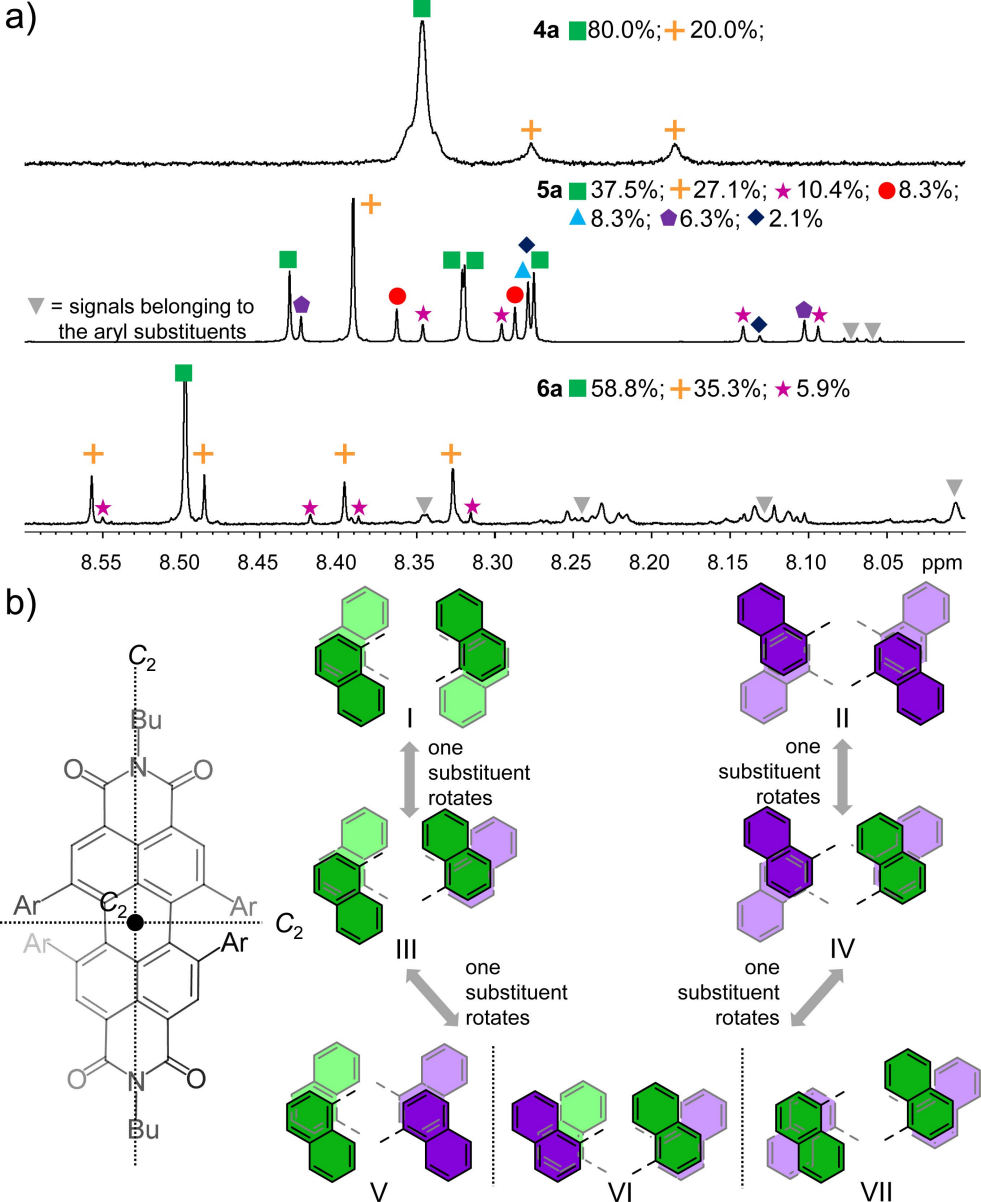
a) Aromatic region of the ^1^H NMR spectra of **4 a** (top, 263 K, TCE‐*d* ^2^), **5 a** (middle, 293 K, CD_2_Cl_2_) and **6 a** (bottom, 293 K, CD_2_Cl_2_) assigned to the *ortho*‐protons of the PBI core as well as ratio of the different conformational isomers found in the respective region; b) possible structures and transformations of the seven conformational isomers for the *P*‐enantiomer of **5 a**.

For the 2‐naphthyl‐functionalized PBI **4 a**, the *T*_c_ (323 K) and the Δ*G* ^≠^ (66.9 kJ mol^−1^) are slightly higher than in **2 a**, which can also be accounted for by an increased *π‐π*‐stacking interaction between the larger substituents (Figure 1a,b). Still, rotations of the interlocked 2‐naphthyl substituents are possible at elevated temperatures, enabling a multitude of different conformations in solution. At lower temperatures (263 K), a second set of signals becomes visible, which is assigned to a less symmetric conformational isomer compared to the conformation responsible for the major signal (see Figure 3a, top, for structural suggestions, see Figure S5). Taking advantage of the molecular structure derived from single crystal X‐ray analysis (Figure 1b) we ascribe the major set of signals for the PBI *ortho*‐protons, a singlet at 8.38 ppm at low temperatures of 263 K, to the highest symmetry conformation I, which exhibits the largest *π‐π*‐overlap of both pairs of neighboring naphthalene units and the least shielded protons in *ortho*‐position of the chromophore core (Figure S5). The smaller set of signals, which is slightly upfield shifted and composed of two singlets of equal size, is ascribed to one of the *C*_2_ symmetric structures V, VI or VII (Figure S5). Based on the presumably favored larger *π*‐overlap provided between two naphthalene subunits in one of the two bay areas we tentatively assigned these peaks to conformational isomer V, which could originate from a concerted rotation of two adjacent 2‐naphthyl substituents starting from the symmetric structure I. According to the sizes of their respective integrals, structures I and V presumably exist at 263 K in CD_2_Cl_2_ in a ratio of 4 : 1 (Figure 3a), while only the dominant one is found in the respective single crystal structure of **4 a** (Figure 1b).

In contrast to **4 a**, upon attachment of four naphthalene units via the 1‐position (**5 a**), a multitude of signal sets is found at room temperature in CD_2_Cl_2_ in the ^1^H NMR for the PBI *ortho*‐protons, which cannot be assigned to the respective conformational isomers in a straightforward manner (Figure 3a, middle). Additionally, no significant differences are observable by variable temperature ^1^H NMR at the lowest and highest temperatures, indicating the formation of either conformationally stably interlocked or easily interchangeable isomers due to the higher steric demand of the substituents in solution (Figure S3). Closer inspection of the aromatic region results again in the assignment of the peaks between 8.50 and 8.08 ppm to the four *ortho*‐protons of the PBI core, as the integral of this area corresponds exceptionally well to four protons, when calibrating the integrals to the alkyl chain in the aliphatic region of the NMR spectrum. By precise integration, seven sets of singlets could be distinguished for these proton signals and assigned to the overall fourteen conformers with varying symmetry that are obviously all present in solution (Figure 3b), considering that the *P*‐ and *M*‐enantiomers cannot be distinguished by ^1^H NMR spectroscopy. A tentative assignment of the peaks was possible by taking into account the symmetry of the respective conformation, the *π*‐overlap of the four aryl‐substituents as well as the shielding and de‐shielding of the *ortho*‐protons by the 1‐naphthyl substituents. Thus, the most intense and isolated singlet at 8.39 ppm (orange cross, Figure 3a) should again correspond to structure I with *D*_2_ symmetry (Figure 3b), while the other highly symmetric conformer II also with three *C*_2_ axes should correspond to the singlet at 8.28 ppm (blue triangle). These structures account to about 27.1 % (I) and 8.3 % (II) of all the present conformations in solution (Figure 3a). The rotation of only one naphthalene substituent from either structure I or II leads to a tremendous loss of symmetry and thus four independent singlets are expected for structures III and IV, as observed in the ^1^H NMR spectrum (green rectangle equal 37.5 %, pink star equal 10.4 %). The subsequent rotation of another naphthalene unit can result in three different conformational isomers V, VI and VII, which all contain again one *C*_2_ axis resulting in two singlets in the ^1^H NMR spectrum. Two sets of signals are identified unambiguously (purple pentagon equal 6.3 %, red circle equal 8.3 %), while the second singlet for the third conformer (dark blue diamond equal 2.1 %) is presumably located beneath other signals at about 8.27 ppm, taking the integral and shapes of the signals as well as expected position into account. The coexistence of all seven conformational isomers for one enantiomer of PBI **5 a** in solution at 293 K in contrast to only two isomers for PBI **4 a** at 263 K might be explained by the larger preference of structure I of the latter due to better stabilization by *π‐π*‐interactions of the adjacent *π*‐stacked substituents (Figure 3b, Figure S5). The large conformational heterogeneity observed for **5 a** in solution might also explain our failure to obtain single crystals suitable for X‐ray crystallography while for **4 a** this was achieved in a straightforward manner.

Interestingly, again in contrast to **5 a** only three different conformationally stable species are observed at room temperature in the ^1^H NMR spectrum of **6 a** in CD_2_Cl_2_, of which the derivative with the highest symmetry is the main conformer (Figure 3a, bottom). This can be rationalized by the even higher sterical demand of the pyrene groups, providing significantly larger interacting *π*‐surfaces and disfavoring the rotation of the aryl substituents. Guided again by the structure found in the solid state (Figure 1c), the derivative with the highest symmetry and highest share of 58.8 % of all conformational isomers should be structure I (Figure 3a, Figure S6). Subsequently, taking into consideration that symmetry breaking upon rotation of one pyrene unit should result in four singlets in the ^1^H NMR spectrum, the other signals for the *ortho*‐protons of the PBI core might be tentatively assigned to conformers III and IV. We like to note that it was not possible for us to further assess the structures by 2D NMR spectroscopy due the proximity of the signals and their very different intensity in the ^1^H NMR spectrum which prevents the attribution of signals to exclusively one proton. Indeed, the small amount of some of these conformational isomers results in signals that are poorly distinguishable from the baseline of the spectra.

To summarize our conformational analysis by NMR studies, the four bay substituents not only result in twisted PBIs bearing conformationally stable *M*‐ and *P*‐atropoenantiomers but may in addition afford the coexistence of up to seven conformational isomers for PBIs **4 a**, **5 a** and **6 a** in solution due to the activated rotation around the respective PBI‐aryl bonds at room temperature. The prevalence of one single conformer and the number and amount of the others could be explained by comparison of the rotational barriers of the aryl substituents, the resulting *π‐π*‐overlap of the neighboring bay substituents as well as sterical and symmetry considerations.

### Absorption and emission properties

**Figure 4:**
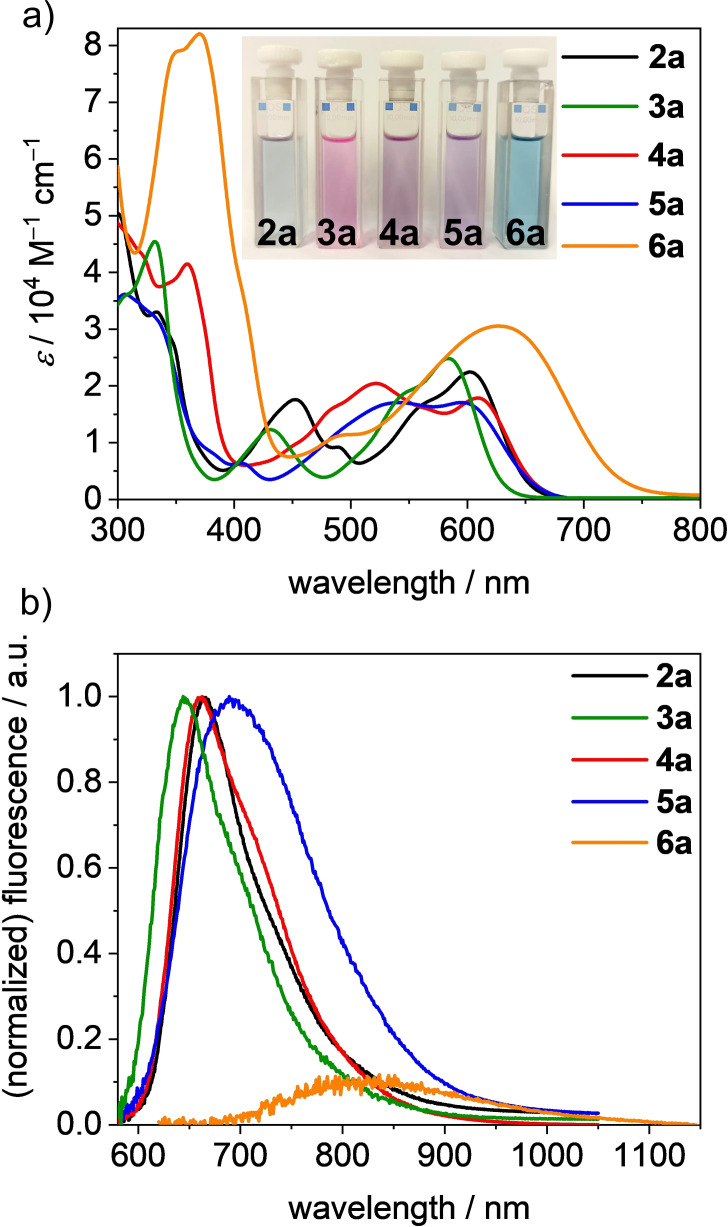
a) UV/Vis/NIR absorption spectra and b) partially normalized emission spectra of PBIs **2 a** (black), **3 a** (green), **4 a** (red), **5 a** (blue) and **6 a** (orange) measured in DCM (*c*_0_=4 ⋅ 10^−4^ M) at room temperature. Inset: Photograph of the solutions **2 a**–**6 a** (left to right, *c*_0_=4 ⋅ 10^−4^ M).

The series of PBI chromophores **2 a**–**6 a** in dichloromethane (DCM) exhibits colors from pink (**3 a**), a reddish blue (**2 a**) over violet (**4 a**, **5 a**) up to turquoise (**6 a**) (Figure 4a, Inset). Their molar absorption coefficients (*ϵ*_max_) in DCM are significantly decreased to 17100–28900 M^−1^ cm^−1^ compared to the unsubstituted *N*,*N’*‐bis(2,6‐diisopropylphenyl)perylene‐3,4 : 9,10‐bis(di‐carboximide) **7** (92900 M^−1^ cm^−1^) and *N*,*N*’‐bis(2,6‐diisopropylphenyl)‐1,6,7,12‐tetraphenoxyperylene‐3,4 : 9,10‐bis(dicarboximide) **8** (48800 M^−1^ cm^−1^).[^30^, ^40^] However, in contrast to these most utilized strongly emissive PBIs, the absorption bands of the tetraaryl‐PBIs are also significantly broadened and bathochromically shifted, thereby reaching into an interesting spectral range. For tetraphenyl‐substituted PBI **2 a**, two major absorption bands are distinguishable in the visible range. The absorption maximum (*λ*_max_) at 602 nm can be assigned to the S_0_‐S_1_ transition of the PBI, while the band at 452 nm is assigned to the S_0_‐S_2_ transition which gains in oscillator strength due to the core twist resulting from the sterical crowding in bay area by the four phenyl groups. The impact of the imide substituent on both spectral shape and position as well as the absorption coefficient is only marginal for **2 b**–**2 e** (see Figure S8). In contrast, the different aryl substituents induce variations in spectral shapes and position of the respective absorption maxima, thereby suggesting some degree of conjugation with the PBI core (Figure 4a). Compared to **2 a**, the absorption maximum of **3 a** is hypsochromically shifted by 19 nm (540 cm^−1^) to 583 nm (24800 M^−1^ cm^−1^) which might be explained by a weaker conjugation due to the more electron withdrawing character of the trifluoromethyl phenyl groups (see below). In case of PBIs **4 a**–**6 a** bearing larger aromatic substituents that no longer exhibit axial symmetry with respect to the connection to the PBI core, no proper separation of the two absorption bands is observable anymore and their spectra are even more broadened than the spectra of **2 a** and **3 a**. The broadening might be attributed to the conformational heterogeneity of the PBI bay substituents (see above), which are a result of the rotation and arrangement of the aryl substituents with respect to each other as well as the twist in bay position. Each conformer exhibits a different absorption spectrum and thus the ensemble spectrum observed by the measurements of solutions results from overlapping spectra of the different conformational isomers identified by our NMR studies. The absorption band of **4 a** and **5 a** between 450 and 650 nm exhibits two poorly distinguishable absorption maxima. DFT calculations suggest the presence of the S_0_‐S_1_ transition as well as higher transitions underneath the main S_0_‐S_1_ absorption band, resulting in a higher overall molar absorptivity (Table S8 and Table S9). Interestingly, in the absorption spectrum of derivative **6 a** bearing four 1‐pyrenyl substituents only one broad and even more bathochromically shifted absorption band is observed. The increased conjugation of these electron‐rich substituents and the electron‐poor perylene core results in a red‐shift of the absorption maximum up to 628 nm which can be attributed to a charge transfer (CT).^[47]^ DFT calculations of the HOMO and the LUMO show a localization of the HOMO mainly on the pyrene units and bay area of the PBI, while the LUMO is localized on the central PBI moiety, supporting the assumption of the charge transfer as source of the broadened absorption band. For all other derivatives, the HOMO is delocalized over the whole molecule including the aryl substituents (Figure S9). Additionally, similar to **5 a**, stable conformational isomers are formed, thereby broadening the absorption spectrum. Below 400 nm, intense absorption bands are present in all spectra. These are commonly assigned to higher energy transitions localized on the respective aryl substituents.

The fluorescence spectra of **2 a**–**6 a** upon excitation at 560 nm (600 nm for **6 a**) are shown in Figure 4b. No vibronic structure is observed for the tetraarylated PBIs in contrast to the emission spectrum of the unsubstituted parent PBI **7**. Further, the Stokes shifts (Δ${\tilde{\nu }}_{Stokes}$ ) of **2 a**–**6 a** are quite large with values between 1200 and 1700 cm^−1^ (Table 1). For **6 a** the Δ${\tilde{\nu }}_{Stokes}$ of 3700 cm^−1^ is even larger. The overall larger Δ${\tilde{\nu }}_{Stokes}$ compared to the parent PBI and other PBI derivatives^[40]^ can be ascribed to the participation of the aryl substituents in the lowest excited state upon electronic and structural relaxation after photoexcitation. The fluorescence quantum yields (*Φ*_F_) are decreasing from 72 % for **3 a**, bearing a rather electron‐withdrawing aryl substituent, to 43 % for **2 a**, 33 % for **4 a**, 15 % for **5 a** and 1 % for **6 a** with the more electron‐rich phenyl, naphthalene and pyrene substituents, respectively. The fluorescence lifetimes (*τ*_F_) range between 9.7 and 12.8 ns for PBIs **2 a**–**5 a** with the exception of **6 a** whose reduced value of 2.2 ns is again attributable to the CT character of the transition. This is also reflected by investigation of the radiative rate constants *k*_r_ that decrease from about 56.3 ⋅ 10^7^ s^−1^ for **3 a** to 15.4 ⋅ 10^7^ s^−1^ for **5 a** and the non‐radiative rate constants *k*_nr_ that increase even more significantly from 21.9 ⋅ 10^7^ s^−1^ to 87.4 ⋅ 10^7^ s^−1^ for **3 a** and **5 a**, respectively. The substitution of the imide position exhibits only a minor influence on the emission spectra, fluorescence lifetimes and fluorescence quantum yields. The values and spectra of **2 a**–**2 e** are almost identical with emission maxima between 663 and 680 nm and comparable shapes of the spectra (Figure S8).

**Table 1:** Summary of the optical and electrochemical properties of compounds **2 a**–**6 a** in DCM at 298 K.

PBI	*λ*_abs_^[a]^ [nm]	*ϵ*_max_ [M^−1^ cm^−1^]	*λ*_em_ [nm]	Δ${\tilde{\nu }}_{Stokes}\;$ [cm^−1^]	*Φ*_F_^[b]^ [%]	*τ*_F_^[c]^ [ns]	*E*_red1_^[d]^ [V]	*E*_red2_^[d]^ [V]	*E*_ox1_^[d]^ [V]	*E*_ox2_^[d]^ [V]	*E*_HOMO_^[e]^ [eV]	*E*_LUMO_^[e]^ [eV]	*E*_gap_ [eV]
**2 a**	602 452	22500 17600	663	1500	43	11.9	−1.05	−1.21	0.97	−^[f]^	−6.12	−4.10	2.02
**3 a**	583 429	24800 12200	644	1600	72	12.8	−0.93	−1.20	1.22	1.53	−6.37	−4.22	2.15
**4 a**	609 521	17900 20400	662	1300	33	11.6	−1.08	−1.26	0.95	−^[f]^	−6.10	−4.07	2.03
**5 a**	596 541	17100 17100	689	1200	15	9.7	−1.07	−1.24	0.98	1.17	−6.13	−4.08	2.05
**6 a**	628 370	28900 80900	816	3700	1	0.6 (47 %) 2.2 (53 %)	−1.04	−1.23	0.69	0.94	−5.84	−4.11	1.73

[a] Spectra were measured in DCM (*c*_0_≈4 ⋅ 10^−6^ M) at room temperature. [b] Fluorescence quantum yields were determined using the dilution method (OD <0.05) and Oxazine 1 (for **2 a**, **4 a**–**6 a**, *Φ*_F_=0.11 in EtOH)^[57]^ or *N*,*N*’‐bis(2,6‐diisopropylphenyl)‐ 1,6,7,12‐tetraphenoxy‐perylene‐3,4 : 9,10‐bis(dicarboximide) (for **3 a**, *Φ*_F_=0.96 in CHCl_3_)^[5]^ as reference. [c] Fluorescence lifetimes were determined with EPL picosecond pulsed diode lasers for time‐correlated single photon counting (*λ*_ex_=505.8 nm). [d] Half‐wave potentials were determined by cyclic or square wave voltammetry measured in DCM (0.1 M TBAHFP) vs. Fc/Fc^+^. [e] Calculated according to literature known procedure using the experimentally determined redox potentials (*E*_HOMO_=−[*E*_ox1_+5.15 eV] and *E*_LUMO_=−[*E*_red1_+5.15 eV]) and the energy level of Fc/Fc^+^ with respect to the vacuum level (−5.15 eV).^[19]^ [f] Not observed.

### Chiroptical properties

Due to the large effective van‐der‐Waals radii of the aryl substituents these tetraaryl‐substituted PBIs afford stable *P*‐ and *M*‐atropoenantiomers with axial chirality.[^30^, ^33^] The separation of the enantiomers has been achieved for PBIs **2 a**, **3 a** and **4 a** by semipreparative HPLC using a column packed with amylose tris‐(3,5‐dimethylphenyl) carbamate immobilized on silica gel.

**Figure 5:**
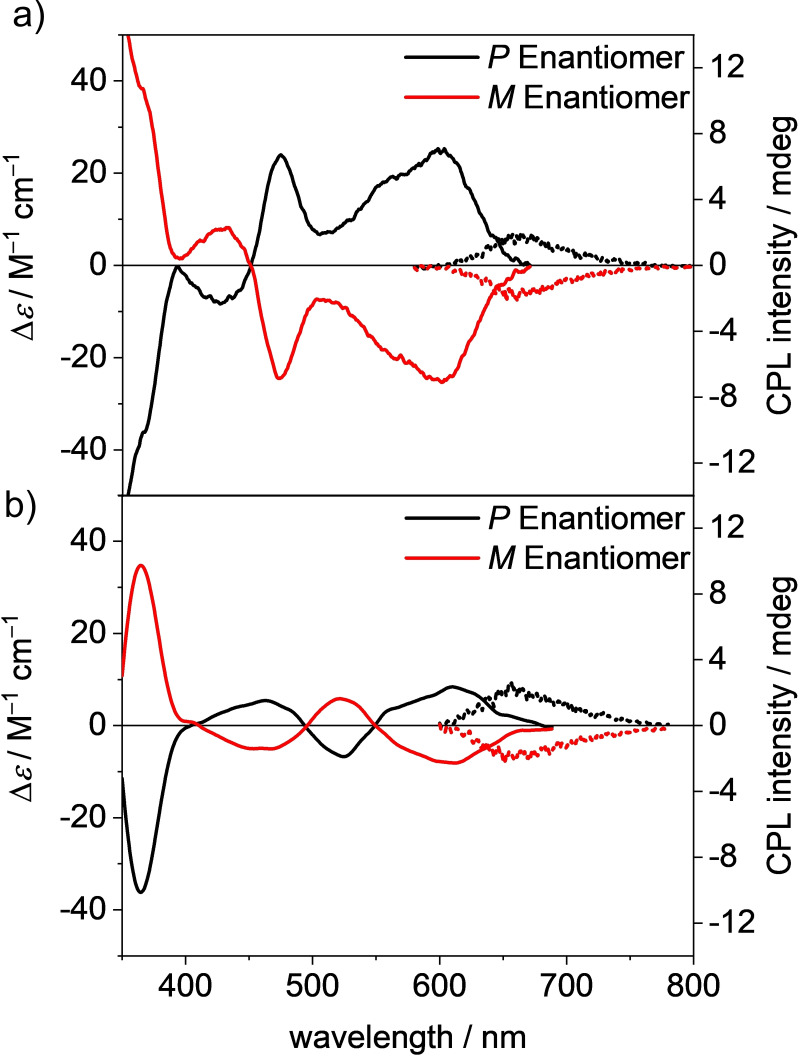
Circular dichroism (CD, *c*_0_=1 ⋅ 10^−5^ M) absorption and circularly polarized luminescence (CPL, *c*_0_=2 ⋅ 10^−6^ M) spectra of *P* (black) and *M* (red) atropoenantiomers of PBIs a) **2 a** and b) **4 a** in DCM at room temperature.

In contrast, the separation of the atropoenantiomers was not possible for derivatives **5 a** and **6 a**, probably due to the numerous conformational isomers present in solution (see above, Figure S11). Circular dichroism (CD) spectra of the separated atropoenantiomers of PBIs **2 a**–**e**, **3 a** and **4 a** were recorded and are shown in Figure 5 and Figure S10. They exhibit a mirror image relation and broad monosignated bands for the S_0_‐S_1_ spectral region (500–600 nm for **2 a**–**e**, **3 a**) and a bisignate Cotton effect for the higher energy S_0_‐S_2_ spectral region (400–500 nm for **2 a**–**e**, **3 a**). The longest wavelength absorption in the visible region between 500 and 600 nm can be assigned to a transition dipole moment polarized along the long molecular *N*,*N*’‐axis, while the second band between 450 and 500 nm can be assigned to a transition polarized along the short molecular axis (the two twisted naphthalene planes).[^30^, ^34^] The enantiomers of **4 a** show bisignate bands for the S_0_‐S_2_ transition band, that are shifted to longer wavelength, thereby relating to the absorption maximum at 521 nm. The enantiomer with the positive Cotton effect for the lowest energy transition is assigned in accordance with our earlier work on tetraphenoxy‐PBIs^[34]^ and theoretical calculations to the *P*‐enantiomer, while the negative signature in the visible region correspond to the *M*‐enantiomer.

The racemization of the *P*‐enantiomers of **2 a** and **4 a** have been investigated by time‐dependent CD measurements, which were performed at four elevated temperatures between 383 and 398 K in 1,1,2,2‐tetrachloroethane (Figure S12, Figure S13, Table S4). Accordingly, the rate constants could be determined from the change of the amplitude of the CD signal at 480 nm for **2 a** and 460 nm for **4 a**, respectively. For both derivatives half‐lifetimes of 59 and 43 min at 383 K were recorded, respectively, which are even longer than the reported half‐lifetime of 36 min at 383 K for the tetrabromo‐substituted PBI.^[33]^ This emphasizes the extraordinary conformational stability of the enantiomers resulting from *π‐π*‐stacked interlocking motif of the aryl substituents revealed from the crystal structures (Figure 1 a–c). The free enthalpies of activation Δ*G* ^≠^ at 383 K were determined to be 119.6 kJ mol^−1^ for **2 a** and 118.6 kJ mol^−1^ for **4 a** respectively, indicating only a minor influence of the size of the aryl substituent when attached via the naphthalene *β*‐position. The values for the free enthalpy of activation of **2 a** and **4 a** are comparable to the ones of hexahelicene (154.3 kJ mol^−1^)^[48]^ or a trisubstituted biphenyl (125.4 kJ mol^−1^)^[49]^ which also exhibit conformationally stable isomers. DFT calculations of the transition state confirmed the high free activation enthalpy (Figure S4a). Furthermore, the free activation enthalpy of the rotation of the phenyl substituents was found to be significantly lower (Figure S4b). This explains that this process could be monitored by ^1^H NMR spectroscopy (Figure S1), while the enthalpy of the racemization is too high to be determined by this technique.

In addition, we were able to study the circular polarized luminescence (CPL) for the enantiomers **2 a** (*λ*_ex_=452 nm), **3 a** (*λ*_ex_=430 nm) and **4 a** (*λ*_ex_=510 nm) (Figure 5, Figure S10e). The *g*_lum_ values of around 1 ⋅ 10^−3^ for the heavily twisted molecules are of typical magnitude and are comparable to those of chiral peropyrene reported by Chalifoux *et al*. and other chiral organic molecules.[^37^, ^50^, ^51^, ^52^, ^53^] The monosignated spectra of the two respective *P*‐/*M*‐enantiomers are again mirror images, and the observed Stokes shifts are in accordance with the Stokes shifts obtained by UV/Vis/NIR and fluorescence measurements. The sign of the CPL spectrum matches the sign of the longest wavelength transition in the CD spectrum. The CPL spectra up to 655 nm for **4 a** correspond well with the PBI emission spectra and are among the most red‐shifted CPL spectra reported to date.^[54]^

### Redox properties

**Figure 6:**
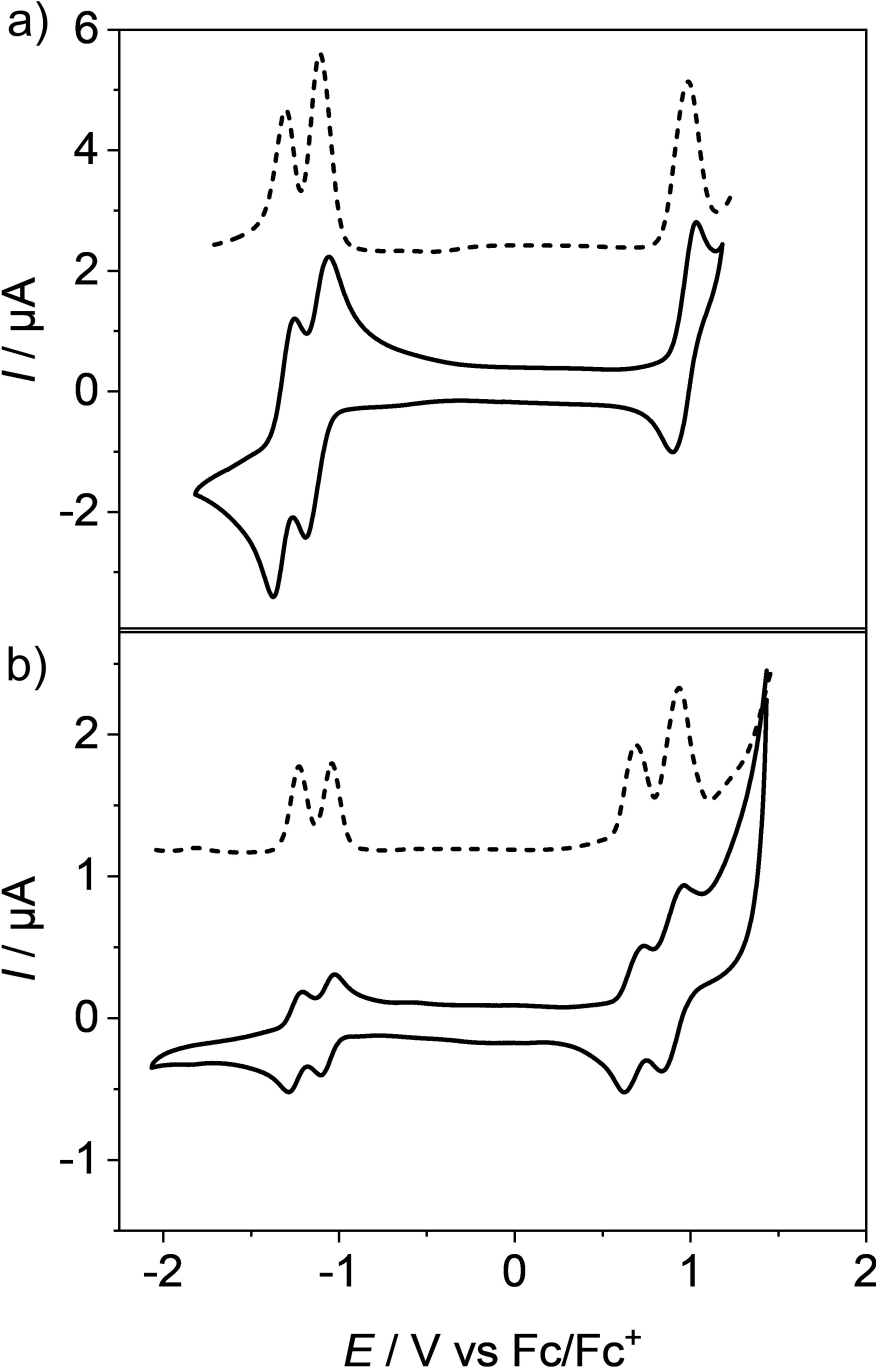
Cyclic voltammograms (solid lines) and square wave voltammograms (dashed lines) of PBIs a) **2 a** and b) **6 a**. Measurements were performed using DCM solutions (*c*_0_=2 ⋅ 10^−4^ M) at 293 K, using TBAHFP (*c*_0_=0.1 M) as electrolyte.

The electronic effect imparted by the different aryl substituents in bay position has been investigated by cyclic voltammetry (CV) and square wave voltammetry (SWV) in DCM with tetrabutylammonium hexafluorophosphate (TBAHFP) as electrolyte. The data obtained from these measurements are summarized in Table 1 and the voltammograms are depicted in Figure 6 and Figure S14 to Figure S17. All investigated PBIs exhibit two reversible reduction waves. With the exception of **3 a**, the first reduction of all derivatives is observed at approximately −1.05 V, while the second reduction occurs at approximately −1.23 V (Figure 6a). These reduction potentials can be attributed to the formation of the respective radical anions and dianions of the PBIs. The comparison to the unsubstituted parent PBI **7** (−1.06 V/−1.26 V) reveals only a minor impact of the aryl substituents on the reduction potentials although the chromophore core is heavily distorted. However, the introduction of *para*‐(trifluoromethyl) phenyl substituents in **3 a** (−0.93 V/−1.20 V) led to a more easy reduction which suggests a stabilization of the negative charge by the electron‐withdrawing substituent. A similar trend is observed upon oxidation of the PBIs. All derivatives with the exception of **6 a**, exhibit at least one reversible oxidation process at approximately +0.97 V, which is comparable to the oxidation potential of tetraphenoxy‐substituted PBIs.^[2]^

In contrast, the first oxidation of PBI **6 a** already occurs at +0.69 V, which is approximately 0.3 V lower than the oxidation of the other PBIs and also with its increased current indicative for a two‐electron process (Figure 6b). As the HOMO of **6 a** is localized mainly on the pyrene subunits (Figure S9) this oxidation can be assigned to two simultaneous one‐electron oxidations (*E*_ox,pyrene_ =+0.63 V, Table S5). If we consider the structural features deduced from the single crystal X‐ray analysis (see above) these observations suggest that each of the two pyrene dimers located on each side of the PBI share one positive charge, i.e., forming so‐called *π*‐dimer cations.[^55^, ^56^] The second two‐electron oxidation at +0.94 V might accordingly be attributed to the second oxidation of the *π*‐dimer upon which each pyrene unit is oxidized. It is noteworthy, however, that at this potential also the perylene core might be oxidized according to studies with the other PBI derivatives.^[2]^

The HOMO and LUMO energy levels included in Table 1 are calculated from the first oxidation and first reduction potential respectively with the energy level of Fc/Fc^+^ set to −5.15 eV vs. vacuum.^[19]^ The HOMOs of all PBIs are located at about −6.10 eV, with the exception of **6 a**, whose HOMO level is approximately 0.4 eV higher in energy. The LUMO energies of the PBIs are approximately −4.10 eV, which render them all suitable candidates as NFAs in OSCs. All HOMO‐LUMO gaps are about 2.00 eV. The only exceptions are **3 a** (enlarged gap of 2.15 eV due to the lower LUMO) and **6 a** (reduced gap of 1.73 eV due to the higher HOMO attributed to the pyrene substituents).

## Conclusion

In conclusion, we synthesized a series of tetraarylated PBIs by Suzuki coupling with variation in the bay as well as the imide positions. These dyes exhibit extraordinarily high solubility in common organic solvents and are readily available due to their straightforward synthesis from easily available precursors. The up to 36.6° twisted structure of these chromophores could for the first time be unambiguously characterized by X‐ray crystallography for three derivatives bearing different aryl substituents which revealed the presence of interlocked *π‐π*‐stacking motifs between these substituents. A complex ensemble of up to fourteen isomers for the PBIs due to rotation of their large bay substituents in solution was observed and assigned by ^1^H NMR spectroscopic analysis. The optical properties were characterized by UV/Vis/NIR and fluorescence spectroscopy, showing compared to the unsubstituted parent PBI reduced molar absorption coefficients but a significant bathochromic shift along with spectral broadening. Depending on the aryl substituent, the fluorescence quantum yield decreases from 72 % for the most electron‐poor arene to about 1 % for the most electron‐rich pyrene substituent. The atropoenantiomers could be separated by HPLC for several derivatives using a chiral stationary phase. Even at elevated temperature their racemization was slow (several hours) according to time‐dependent CD measurements. Circularly polarized luminescence of tetraarylated PBIs was investigated as well, revealing one of the most bathochromic shifted CPL spectra reported to date for small organic molecules.

## Conflict of interest

The authors declare no conflict of interest.

## Supporting information

As a service to our authors and readers, this journal provides supporting information supplied by the authors. Such materials are peer reviewed and may be re‐organized for online delivery, but are not copy‐edited or typeset. Technical support issues arising from supporting information (other than missing files) should be addressed to the authors.

Supporting Information
