# Supplementary material for: Chiral Perylene Bisimide Dyes by Interlocked Arene Substituents in the Bay Area
Source: Chemistry. 2021 Jul 7;27(46):11997–2006. doi: 10.1002/chem.202101877 (PMC8456824; doi:10.1002/chem.202101877)
Supplement: Supplementary file 1 — Supporting Information [file CHEM-27-11997-s001.pdf]

Chemistry–A European Journal

Supporting Information

Chiral Perylene Bisimide Dyes by Interlocked Arene Substituents in the Bay Area

Rebecca Renner, Bernhard Mahlmeister, Olga Anhalt, Matthias Stolte, and Frank Würthner*

Table of Contents

1. Experimental Part S2

1.1. General Methods S2

1.2.1. Synthesis of **2a** S4

1.2.2. Synthesis of **2b** S4

1.2.3. Synthesis of **2c** S5

1.2.4. Synthesis of **2d** S5

1.2.5. Synthesis of **2e** S6

1.2.6. Synthesis of **3a** S7

1.2.7. Synthesis of **4a** S7

1.2.8. Synthesis of **5a** S8

1.2.9. Synthesis of **6a** S9

2. Structural Analysis S10

3. Optical and Electrochemical Characterization S15

4. NMR spectra S27

5. Supporting References S38

1. Experimental Part

1.1. General Methods

Unless otherwise stated, all chemicals, reagents and solvents were purchased from commercial suppliers and used after appropriate purification. The 1,6,7,12-tetrachloroperylene bis(dicarboximides) **1a** - **e** were synthesized according to literature.[S1] Toluene was of HPLC grade and dried prior to use by an Innovative Technology PureSolv solvent purification system. Dichloromethane was distilled prior to use. Column chromatography was performed with commercial glass columns using silica gel 60 M (particle size 0.04 – 0.063 mm; Merck KGaA) as stationary phase. Normal phase HPLC was performed on a Japan Analytical Industry (JAI) recycling preparative HPLC system *LC-9105* equipped with a VP 250/21 NUCLEOSIL 100-7 column of Macherey-Nagel. For the chiral separation of the enantiomers, the same HPLC system was equipped with a Reprosil 100 Chiral-NR 8 μm column from *Trentec*. NMR spectra were recorded on a Bruker *Avance III HD* 400 MHz NMR spectrometer and are calibrated to the residual proton signal of the used deuterated solvent. The chemical shifts (δ) are reported in parts per million (ppm). Multiplicities for proton signals are abbreviated as s, bs, d, bd, t, sep, m and bm for singlet, broad singlet, doublet, broad doublet, triplet, septet, multiplet and broad multiplet respectively. MALDI-TOF mass spectra were recorded with a Bruker Daltonics GmbH *ultrafleXtreme* mass spectrometer using DCBT (2-[(2*E*)-3-(4-*tert*-butylphenyl)-2-methylprop-2-enylidene]malononitrile) as matrix. High resolution mass spectra were measured by electrospray ionization (ESI) with an *ESI microTOF Focus* mass spectrometer from Bruker Daltonics GmbH. The melting points were determined using a SMP50 from *Stuart*. UV/Vis/NIR absorption spectra were recorded on a Jasco V-770 or Jasco V-670 spectrometer. Fluorescence spectra were recorded on a *FLS980* fluorescence spectrometer (Edinburgh Instruments) and were corrected against the photomultiplier sensitivity and the lamp intensity. CD spectra were recorded on a Jasco *J-810*

spectropolarimeter. CPL spectra were recorded with a customised JASCO CPL-300/J-1500 hybrid spectrometer. Cyclic voltammetry measurements were conducted on an *EC epsilon* (BASi instruments, UK) potentiostat connected to a three-electrode single-compartment cell. A Pt disc electrode was used as working electrode, a platinum wire as counter electrode and an Ag/AgCl reference electrode. The spectra were referenced using the ferrocenium/ferrocene redox couple as an internal standard. Single crystal X-ray diffraction data were collected at 100 K on a Bruker D8 Quest Kappa diffractometer with a Photon II CPAD detector and multi-layered mirror monochromated CuK_α radiation. The structures were solved using direct methods, expanded with Fourier techniques and refined with the Shelx software package.[S2] All non-hydrogen atoms were refined anisotropically. Hydrogen atoms were included in the structure factor calculation on geometrically idealized positions. Crystallographic data have been deposited with the Cambridge Crystallographic Data Centre as supplementary publication no. 2086018 (**2c**), 2086019 (**4a**) and 2086020 (**6a**). These data can be obtained free of charge from The Cambridge Crystallographic Data Centre via www.ccdc.ac.uk/data.request/cif. Theoretical calculations were performed by the Gaussian software[S3] using B3LYP/6-31G(d) level of theory for structure optimization and B3LYP/def2-SVP level of theory for TD-DFT simulation of electron transitions.

1.2. General Procedure for the synthesis of the tetraarylated PBI derivatives

The respective 1,6,7,12-tetrachloroperylene bis(dicarboximide) (100 µmol), the arylboronic acid (2.00 mmol, 20 eq.), anhydrous potassium carbonate (145 mg, 1.05 mmol, 10.5 eq.) and tetrakis(triphenylphosphine)palladium (34.7 mg, 30.0 µmol, 0.3 eq.) were added to a Schlenk tube. Toluene (3.2 mL), ethanol (0.6 mL) and water (1.6 mL), which were degassed prior to use, were added and the solution was subjected to three freeze-pump-thaw cycles. After stirring overnight at 80 °C, the mixture was extracted with dichloromethane and washed three times

with water. The organic phases were combined, and the solvent removed under reduced pressure. The crude product was purified by column chromatography and HPLC.

1.2.1. Synthesis of 2a

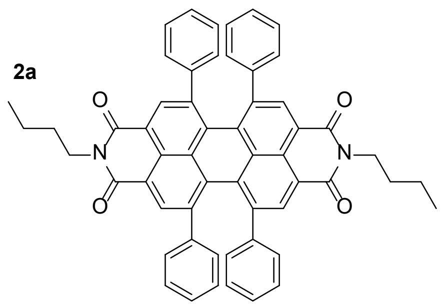

Solvent column chromatography and HPLC: hexane/dichloromethane = 1/3; Yield: 58.9 mg (73.0 mmol, 73%) of a blue-purple solid. ^{1}H NMR (400 MHz, CD_2Cl_2, 293 K): δ = 8.24 (s, 4H), 7.22 (t, 4H), 7.08 (bs, 8H), 6.70 (bs, 8H), 4.18 (t, 4H), 1.74 (m, 4H), 1.47 (m, 4H), 1.00 (t, 6H); ^{13}C-NMR (100 MHz, CD_2Cl_2, 293 K): δ = 163.9, 142.7, 140.9, 133.6, 132.3, 131.7, 129.2, 127.9, 126.4, 122.8, 40.6, 30.7, 20.8, 14.1; MS (MALDI-TOF, pos. mode, DCTB in $CHCl_3$): *m/z* calculated for $C_{56}H_{42}N_2O_4^+$ $[M]^+$: 806.31, found: 806.30; HRMS (ESI-TOF, pos. mode, MeCN/$CHCl_3$): *m/z* calculated for $C_{56}H_{42}N_2NaO_4^+$ $[M+Na]^+$: 829.3037, found 829.3040; m.p. >390 °C.

1.2.2. Synthesis of 2b

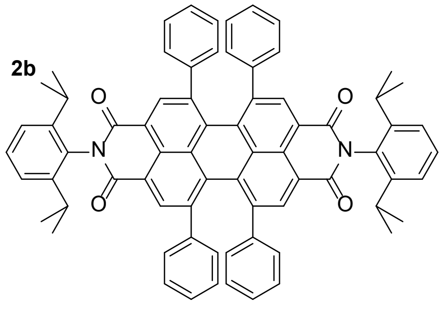

Solvent column chromatography and HPLC: hexane/dichloromethane = 1/3; Yield: 75.8 mg (74.6 mmol, 75%) of a dark blue-purple solid. ^{1}H NMR (400 MHz, CD_2Cl_2, 293 K): δ = 8.35 (s, 4H), 7.53 (t, 2H), 7.38 (d, 4H), 7.26 (m, 4H), 7.15 (bs, 8H), 6.83 (bs, 8H), 2.87 (sep, 4H),

1.22 (d, 12H), 1.15 (d, 12H); ^{13}C-NMR (100 MHz, CD_2Cl_2, 293 K): δ = 166.1, 148.4, 145.3, 142.9, 136.3, 134.6, 134.1, 133.5, 133.0, 131.8, 131.1, 130.0, 129.0, 126.4, 124.5, 31.4, 26.1, 26.05; MS (MALDI-TOF, pos. mode, DCTB in $CHCl_3$): *m/z* calculated for $C_{78}H_{58}N_2O_4^+$ $[M]^+$: 1014.43, found: 1014.40; HRMS (ESI-TOF, pos. mode, MeCN/$CHCl_3$): *m/z* calculated for $C_{78}H_{58}N_2NaO_4^+$ $[M+Na]^+$: 1037.4289, found 1037.4310; m.p. >390 °C.

1.2.3. Synthesis of 2c

2c

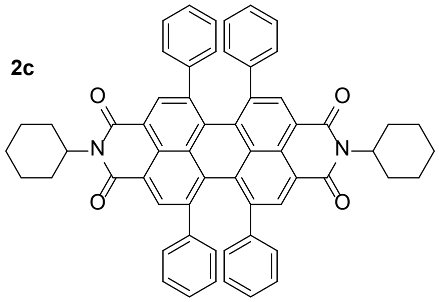

Solvent column chromatography and HPLC: hexane/dichloromethane = 1/3; Yield: 56.6 mg (65.9 mmol, 66%) of a dark black-purple solid. ^{1}H NMR (400 MHz, CD_2Cl_2, 293 K): δ = 8.22 (s, 4H), 7.20 (m, 4H), 7.08 (bs, 8H), 6.70 (bs, 8H), 5.04 (m, 2H), 2.56 (m, 4H), 1.90 (m, 4H), 1.75 (m, 4H), 1.46 (m, 4H), 1.34 (m, 4H); ^{13}C-NMR (100 MHz, CD_2Cl_2, 293 K): δ = 164.3, 142.6, 141.0, 133.5, 132.0, 131.6, 129.0, 127.8, 126.4, 123.3, 32.0, 29.6, 29.5, 27.0, 25.9, 23.0, 14.3; MS (MALDI-TOF, pos. mode, DCTB in $CHCl_3$): *m/z* calculated for $C_{60}H_{46}N_2O_4^+$ $[M]^+$: 858.35, found: 858.34; HRMS (ESI-TOF, pos. mode, MeCN/$CHCl_3$): *m/z* calculated for $C_{60}H_{46}N_2NaO_4^+$ $[M+Na]^+$: 881.3350, found 881.3339; m.p. >390 °C.

1.2.4. Synthesis of 2d

2d

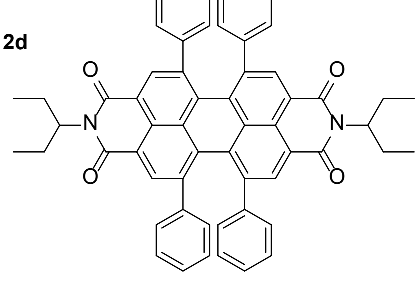

Solvent column chromatography and HPLC: hexane/dichloromethane = 1/3; Yield: 38.1 mg (45.6 mmol, 46%) of a dark purple solid. ^{1}H NMR (400 MHz, CD_2Cl_2, 293 K): δ = 8.23 (s, 4H), 7.20 (m, 4H), 7.10 (bs, 8H), 6.72 (bs, 8H), 5.05 (m, 2H), 2.25 (m, 4H), 1.94 (m, 4H), 0.95 (m, 12H); ^{13}C-NMR (100 MHz, CD_2Cl_2, 293 K): δ = 142.8, 141.0, 133.7, 132.1, 131.7, 130.7, 129.0, 127.7, 126.6, 122.8, 57.8, 25.44, 25.40, 11.56, 11.55; MS (MALDI-TOF, pos. mode, DCTB in $CHCl_3$): *m/z* calculated for $C_{58}H_{46}N_2O_4^+$ $[M]^+$: 834.35, found: 834.37; HRMS (ESI-TOF, pos. mode, MeCN/$CHCl_3$): *m/z* calculated for $C_{58}H_{46}N_2NaO_4^+$ $[M+Na]^+$: 857.3350, found 857.3317; m.p. >390 °C.

1.2.5. Synthesis of 2e

2e

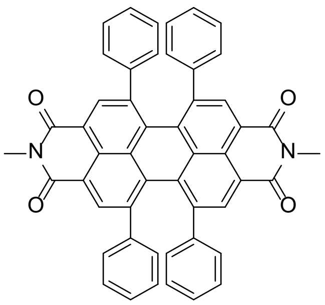

Solvent column chromatography and HPLC: dichloromethane/ethylacetate = 99/1; Yield: 36.6 mg (50.6 mmol, 51%) of a dark black-purple solid. ^{1}H NMR (400 MHz, CD_2Cl_2, 293 K): δ = 8.24 (s, 4H), 7.19 (bs, 4H), 7.06 (bs, 8H), 6.65 (bs, 8H), 3.63 (s, 6H); ^{13}C-NMR (100 MHz, CD_2Cl_2, 293 K): δ = 142.5, 140.6, 133.4, 132.2, 131.6, 131.0, 129.0, 127.8, 126.4, 123.3, 27.1; MS (MALDI-TOF, pos. mode, DCTB in $CHCl_3$): *m/z* calculated for $C_{56}H_{42}N_2O_4^+$ $[M]^+$: 722.22, found: 722.19; HRMS (ESI-TOF, pos. mode, MeCN/$CHCl_3$): *m/z* calculated for $C_{56}H_{43}N_2NaO_4^+$ $[M+Na]^+$: 745.2098, found 745.2093; m.p. >390 °C.

1.2.6. Synthesis of 3a

3a

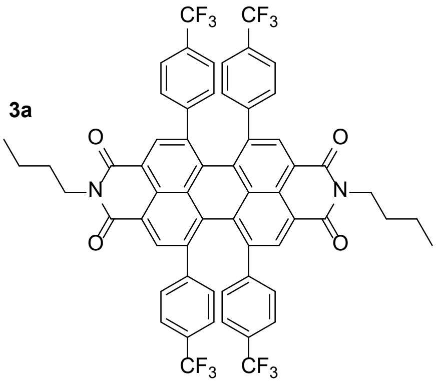

Solvent column chromatography and HPLC: hexane/dichloromethane = 1/3; Yield: 65.1 mg (60.3 mmol, 60%) of a-purple solid. ^{1}H NMR (400 MHz, CD_2Cl_2, 293 K): δ = 8.33 (s, 4H), 7.39 (bs, 8H), 6.88 (bd, 8H), 4.21 (t, 4H), 1.75 (m, 4H), 1.48 (m, 4H), 1.01 (t, 6H); ^{13}C-NMR (100 MHz, CD_2Cl_2, 293 K): δ = 163.4, 144.2, 141.2, 133.8, 131.8, 131.6, 130.1, 129.7, 127.2, 125.8, 123.7, 40.8, 30.7, 20.8, 14.1; MS (MALDI-TOF, pos. mode, DCTB in $CHCl_3$): *m/z* calculated for $C_{60}H_{38}N_2O_4^+$ $[M]^+$: 1078.26, found: 1078.30; HRMS (ESI-TOF, pos. mode, MeCN/$CHCl_3$): *m/z* calculated for $C_{60}H_{38}N_2NaO_4^+$ $[M+Na]^+$: 1101.2532, found 1101.2589; m.p. >390 °C.

1.2.7. Synthesis of 4a

4a

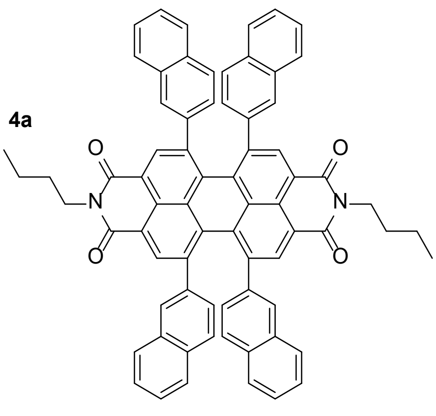

Solvent column chromatography and HPLC: hexane/dichloromethane = 1/3; Yield: 28.3 mg (28.1 mmol, 28%) of a purple solid. ^{1}H NMR (400 MHz, CD_2Cl_2, 293 K): δ = 8.37 – 6.61 (bm, 32H), 4.03 (bs, 4H), 1.71 (bs, 4H), 1.45 (m, 4H), 1.00 (t, 6H); ^{1}H-NMR (400 MHz, TCE-d_2,

390 K): δ = 8.38 (s, 4H), 7.70 (d, 4H), 7.43 (t, 4H). 7.31 (bm, 8H), 6.90 (bm, 8H), 6.63 (d, 4H), 4.23 (t, 4H), 1.80 (m, 4H), 1.50 (m, 4H), 1.02 (t, 6H); ^{13}C-NMR (100 MHz, CD_2Cl_2, 293 K): δ = 163.9, 142.5, 138.2, 135.0, 134.0, 132.5, 132.2, 131.8, 128.4, 127.9, 127.4, 126.9, 126.5, 123.1, 40.5, 30.6, 20.8, 14.1; MS (MALDI-TOF, pos. mode, DCTB in $CHCl_3$): *m/z* calculated for $C_{72}H_{50}N_2O_4^+$ $[M]^+$: 1006.38, found: 1006.36; HRMS (ESI-TOF, pos. mode, MeCN/$CHCl_3$): *m/z* calculated for $C_{72}H_{50}N_2NaO_4^+$ $[M+Na]^+$: 1029.3663, found 1029.3660; m.p. >390 °C.

1.2.8. Synthesis of 5a

5a

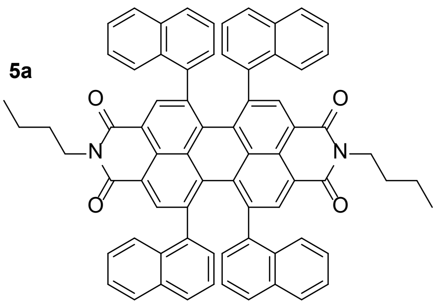

Solvent column chromatography and HPLC: hexane/dichloromethane 1/3; Yield: 37.8 mg (37.5 mmol, 38%) of a purple solid; ^{1}H-NMR (400 MHz, CD_2Cl_2, 293 K): δ = 8.42 – 6.14 (m, 32H), 4.12 (m, 4H), 1.71 (m, 4H). 1.44 (m, 4H), 0.97 (m, 6H); ^{13}C-NMR (100 MHz, CD_2Cl_2, 293 K): δ = 164.0, 163.8, 142.3, 141.9, 141.0, 140.6, 139.9, 139.8, 139.2, 139.0, 138.9, 138.8, 137.2, 136.9, 135.4, 135.1, 135.0, 134.9, 134.8, 134.6, 134.5, 134.0, 133.96, 133.91, 133.8, 133.6, 132.4, 131.9, 131.2, 131.2, 131.1, 131.0, 130.9, 129.9, 129.6, 129.4, 129.2, 128.9, 128.8, 128.5, 128.4, 128.3, 126.8, 126.7, 126.5, 126.4, 126.1, 125.92, 125.88, 125.79, 125.7, 125.5, 125.3, 125.2, 124.4, 124.3, 123.4, 123.3, 123.2, 121.7, 121.6, 121.4, 40.6, 30.6, 20.8, 14.0; MS (MALDI-TOF, pos. mode, DCTB in $CHCl_3$): *m/z* calculated for $C_{72}H_{50}N_2O_4^+$ $[M]^+$: 1006.38, found: 1006.38; HRMS (ESI-TOF, pos. mode, MeCN/$CHCl_3$): *m/z* calculated for $C_{72}H_{50}N_2NaO_4^+$ $[M+Na]^+$: 1029.3663, found 1029.3648; m.p. >390 °C.

1.2.9. Synthesis of 6a

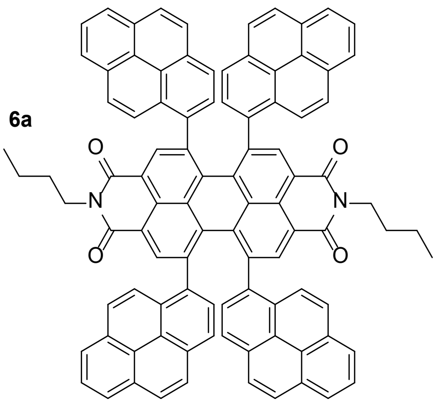

Solvent column chromatography and HPLC: hexane/dichloromethane 1/3; Yield: 28.1 mg (21.6 mmol, 22%) of a blue solid; ^{1}H-NMR (400 MHz, CD_2Cl_2, 293 K): δ = 8.55 – 5.93 (m, 40H), 4.15 (m, 4H), 1.75 (m, 4H), 1.45 (m, 4H), 0.99 (m, 6H); ^{13}C-NMR (100 MHz, CD_2Cl_2, 293 K): δ = 164.1, 142.5, 137.0, 136.0, 134.3, 134.23, 134.18, 132.3, 130.7, 130.6, 130.2, 129.0, 128.7, 127.4, 127.0, 126.2, 125.8, 125.6, 125.2, 125.0, 123.6, 122.1, 121.6, 40.7, 30.7, 20.8, 14.1; MS (MALDI-TOF, pos. mode, DCTB in $CHCl_3$): *m/z* calculated for $C_{96}H_{59}N_2O_4^+$ $[M+H]^+$: 1302.44, found: 1302.53; HRMS (ESI-TOF, pos. mode, MeCN/$CHCl_3$): *m/z* calculated for $C_{96}H_{58}N_2NaO_4^+$ $[M+Na]^+$: 1325.4289, found 1325.4214; m.p. >390 °C.

2. Structural Analysis

Table S1 Structural parameters found in the single crystal structures of **2c**, **4a** and **6a**.

PBI	Space Group	Core twist[a] [°]	*Θ* (C1-C12b-C12a-C12)[b] [°]	*Θ* (C13a-C12a-C12b-C13b)[b] [°]	*Θ* (C12b-C1-C1'-C2')[b,c] [°]
**2c**	P2/c	34.3	32.7/33.4	33.2/33.6	42.1/42.1/43.1/43.1
**4a**	P–1	36.6	33.5/33.5	34.1/35.4	39.4/41.2/41.2/43.3
**6a**	P–1	29.5	29.2/29.9	27.7/29.1	46.6/50.2/54.6/56.9

[a] Angle between the two naphthalene subunits of the PBI; [b] Numbering of carbon atoms according to Scheme 1; [c] For **4a** the angle was measured between C12b-C1-C2'-C3'.

Table S2 Crystallographic data for compounds **2c**, **4a** and **6a**.

	Compound **2c**	Compound **4a**	Compound **6a**
CCDC Number	2086018	2086019	2086020
Empirical Formula	$C_{60}H_{46}N_2O_4$	$C_{72}H_{50}N_2O_4$	$C_{96}H_{58}N_2O_4$
Formula weight / g mol^{-1}	859.04	1007.20	1303.53
Crystal size / mm	0.295 x 0.2687 x 0.192	0.132 x 0.108 x 0.044	0.180 x 0.072 x 0.020
Measurement Temperature	100(2) K	100(2) K	100(2) K
Crystal system	monoclinic	triclinic	triclinic
Space group	C2/c	P-1	P-1
Lattice parameters			
a /Å	11.0037(3)	13.6566(11)	15.5878(5)
b / Å	22.5842(7)	14.4337(12)	15.6577(5)
c / Å	21.5026(6)	17.9111(15)	16.4702(5)
α / °	90	93.237(4)	70.229(2)
β / °	101.5000(10)	105.997(4)	68.009(2)
γ / °	90	94.521(4)	61.8400(10)
Volume/ Å^3	5236.3(3)	3371.7(5)	3221.40(19)
Z value	4	2	2

Calculated Density / g m^{-3}	1.337	1.103	1.344
F(000)	2196	1172	1360
Number of reflections measured	39057	50760	63653
Number of unique reflections	5171	13160	12693
Theta range for data collection	3.915 ° to 72.295 °	2.575 ° to 72.593 °	2.952 ° to 72.267 °
Completeness	100%	99.6%	99.9%
R_{int}	0.0243	0.0321	0.0448
Minimum and maximum transmission	0.5924/0.7536	0.6806/0.7536	0.5985/0.7536
Goodness of fit indicator	1.059	1.056	1.035
Final R_1 indices [I > 2σ(I)] (R_{obs}, wR_{all})	0.0418, 0.1114	0.0639, 0.1615	0.0508, 0.1343
R indices [all data] (R_{obs}, wR_{all})	0.0425, 0.1120	0.0718, 0.1670	0.0652, 0.1438
Largest diff peak and hole (e·Å^{-3})	0.410/–0.289	0.522/–0.414	0.482/–0.432

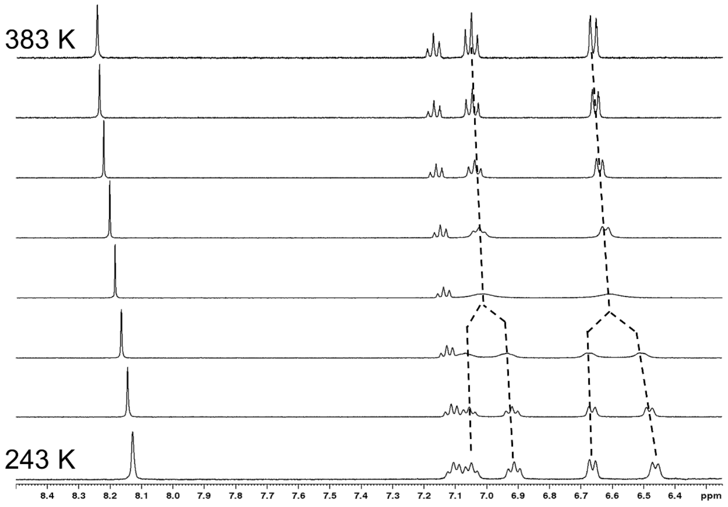

Figure S1 Aromatic region of ^{1}H-NMR spectra of **2a**, measured in 1,1,2,2-tetrachloroethane-d^2 at different temperatures in steps of 20 K from 243 K (bottom) to 383 K (top).

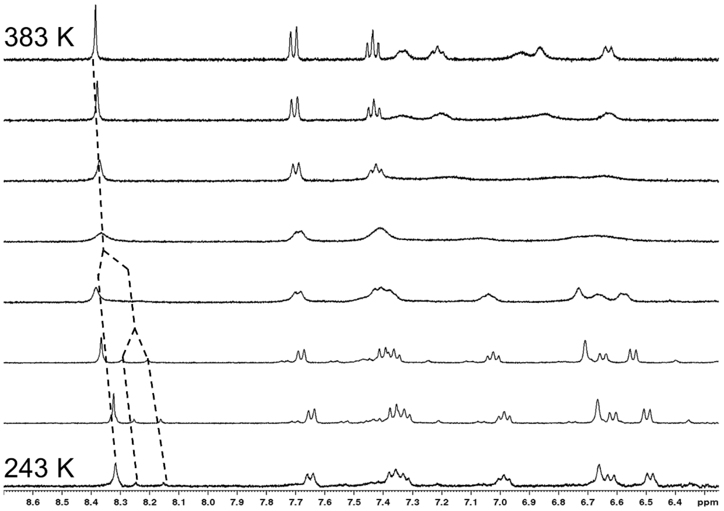

Figure S2 Aromatic region of ^{1}H-NMR spectra of **4a**, measured in 1,1,2,2-tetrachloroethane-d^2 at different temperatures in steps of 20 K from 243 K (bottom) to 383 K (top).

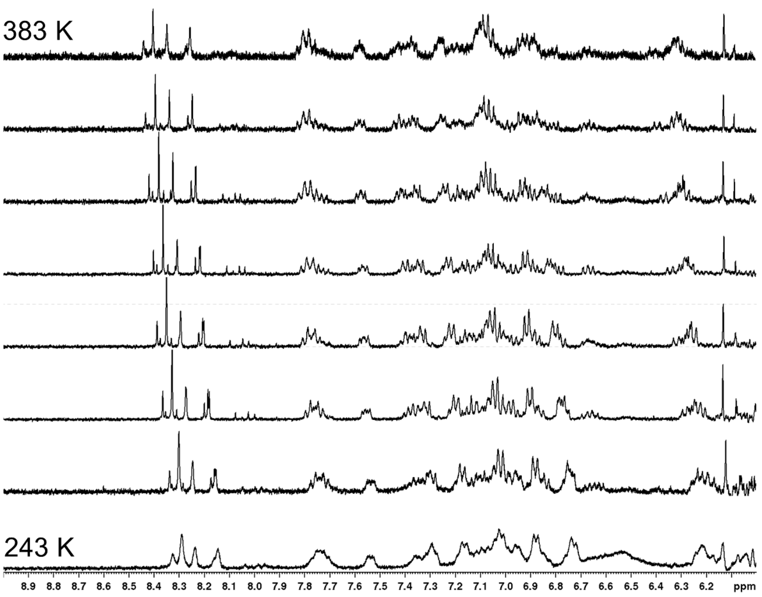

Figure S3 Aromatic region of ^{1}H-NMR spectra of **5a**, measured in 1,1,2,2-tetrachloroethane-d^2 at different temperatures in steps of 20 K from 243 K (bottom) to 383 K (top).

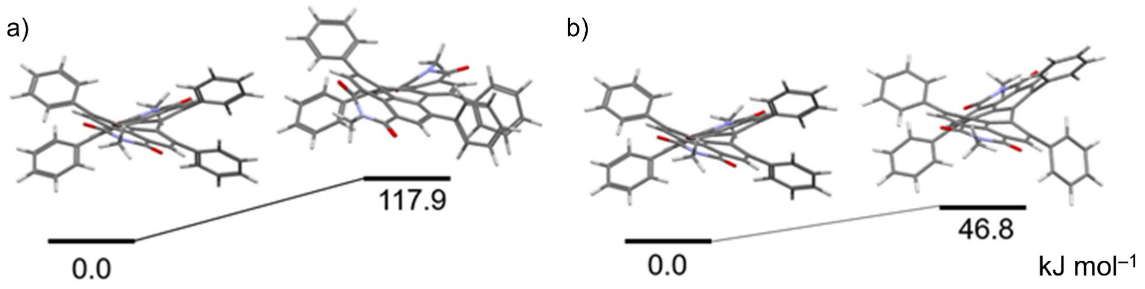

Figure S4 Relative energies in kJ mol^{-1} of the transition states of the a) *M*/*P*-racemization and b) rotation of one phenyl ring relative to the ground state energies of the *M*-enantiomer of PBI derivative **2e** calculated by the Gaussian 09 program using DFT B3LYP/6-31G (d).

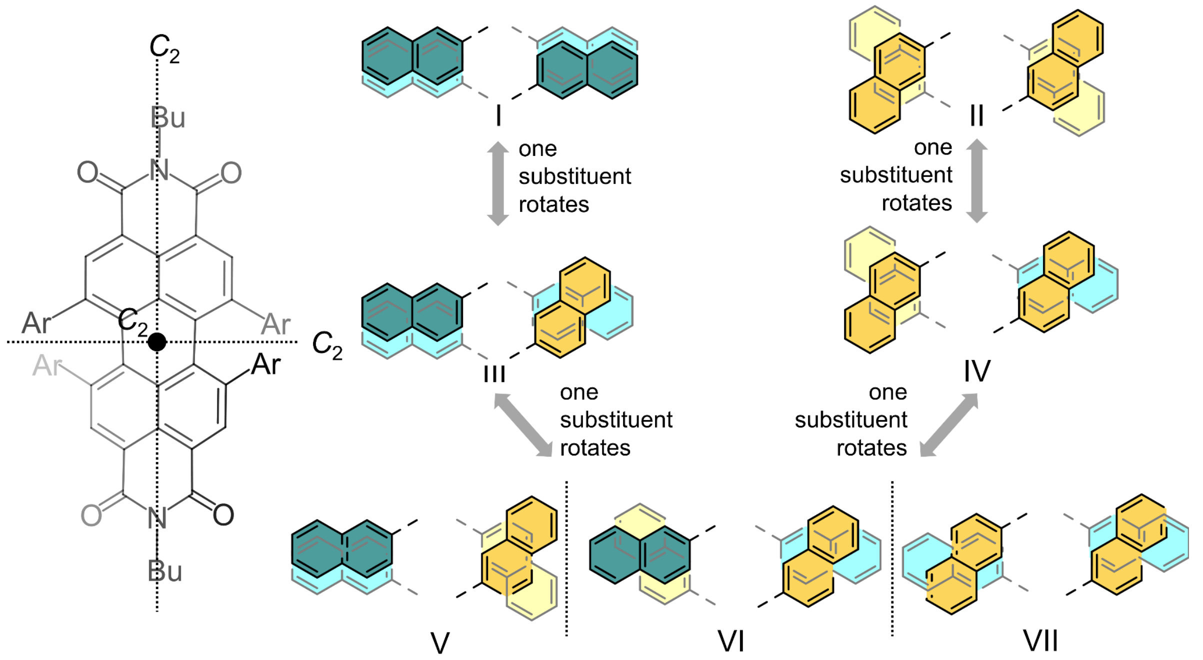

Figure S5 Possible arrangements of the four 2-naphthalene substituents in the bay-region of the *P*-enantiomer of **4a**.

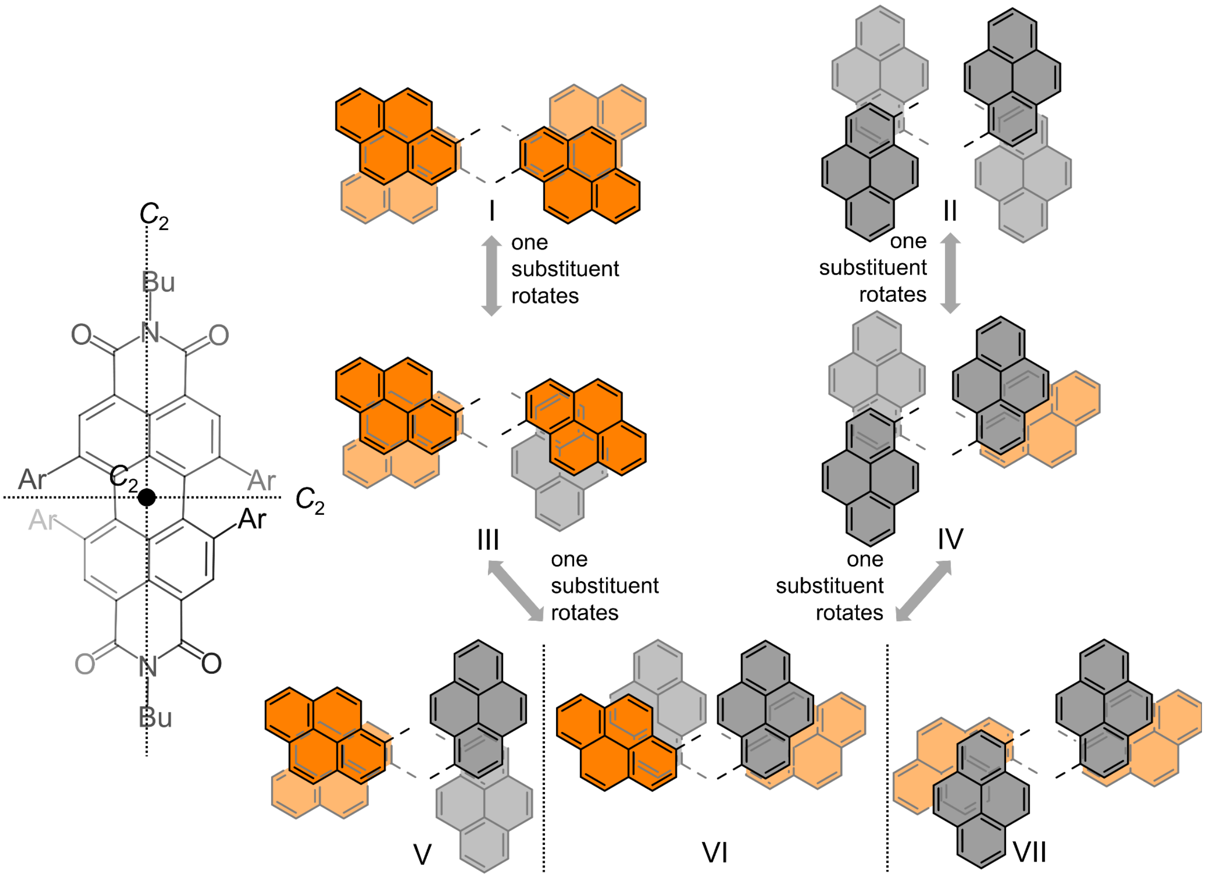

Figure S6 Possible arrangements of the four 1-pyrene substituents in the bay-region of the *P*-enantiomer of **6a**.

3. Optical and Electrochemical Characterization

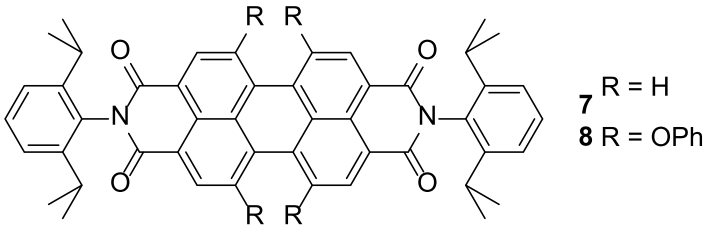

Figure S7 Chemical structures of reference compounds **7** and **8**.

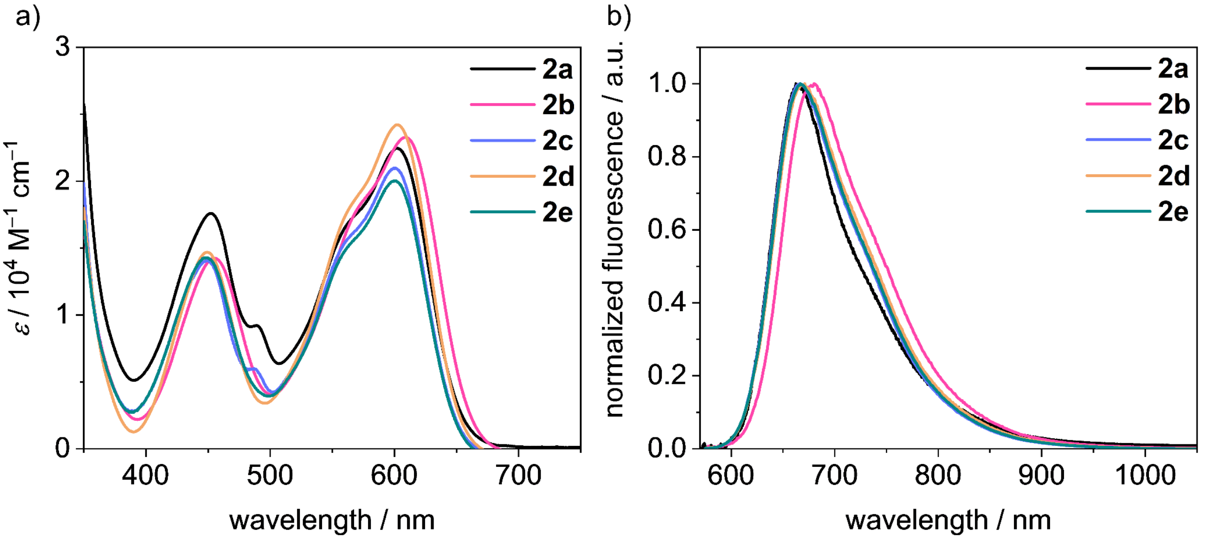

Figure S8 a) UV/Vis/NIR absorption spectra of **2a**-**2e** as well as b) normalized emission spectra measured in DCM ($c_0 \approx 4{\cdot}10^{-6}$ M) at 293 K.

Table S3 Summary of the optical properties of **2a**-**2e** measured in DCM at 293 K.

PBI	λ_{abs}[a] [nm]	ε_{max} [M^{-1} cm^{-1}]	λ_{em} [nm]	$\tilde{\nu}_{Stokes}$ [cm^{-1}]	Φ_f[b] [%]	τ_F[c] [ns]
**2a**	602<br>452	22500<br>17600	663	1530	43	11.9
**2b**	609<br>455	23300<br>14300	680	1690	46	10.9
**2c**	601<br>449	21000<br>14100	666	1620	49	12.0
**2d**	602<br>449	24200<br>14700	671	1740	55	11.7
**2e**	601<br>448	20000<br>14300	667	1650	41	12.3
**7**	527<br>491	93000<br>60800	534	210	99	3.7
**8**	576<br>444	48800<br>16500	608	920	96	6.0
Pyrene	337<br>322	34400<br>23400	372[d,S4]	700[d,S4]	64[d,S4]	354[d,S4]

[a] Spectra were measured in dichloromethane ($c_0 \approx 4\cdot10^{-6}$ M) at 293 K. [b] Fluorescence quantum yields were determined using the dilution method (OD < 0.05) with Oxazine 1 in ethanol (Φ_f = 11%) as standard.[S5] [c] Fluorescence lifetimes were determined with EPL picosecond pulsed diode lasers (λ_{ex} = 560 nm) for time correlated single photon counting; [d] in hexane.

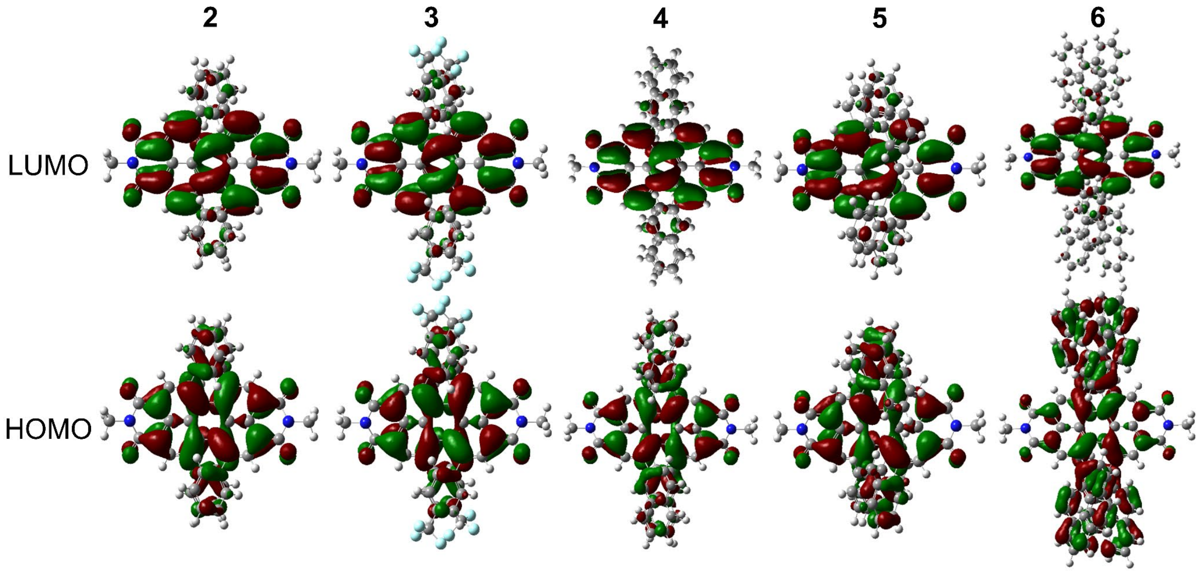

Figure S9 Calculated HOMO (bottom) and LUMO (top, isovalue = 0.2 a.u.) of PBIs **2** - **6** at B3LYP/6-31g(d) level theory. The imide-substituents have been replaced by methyl groups for simplicity.

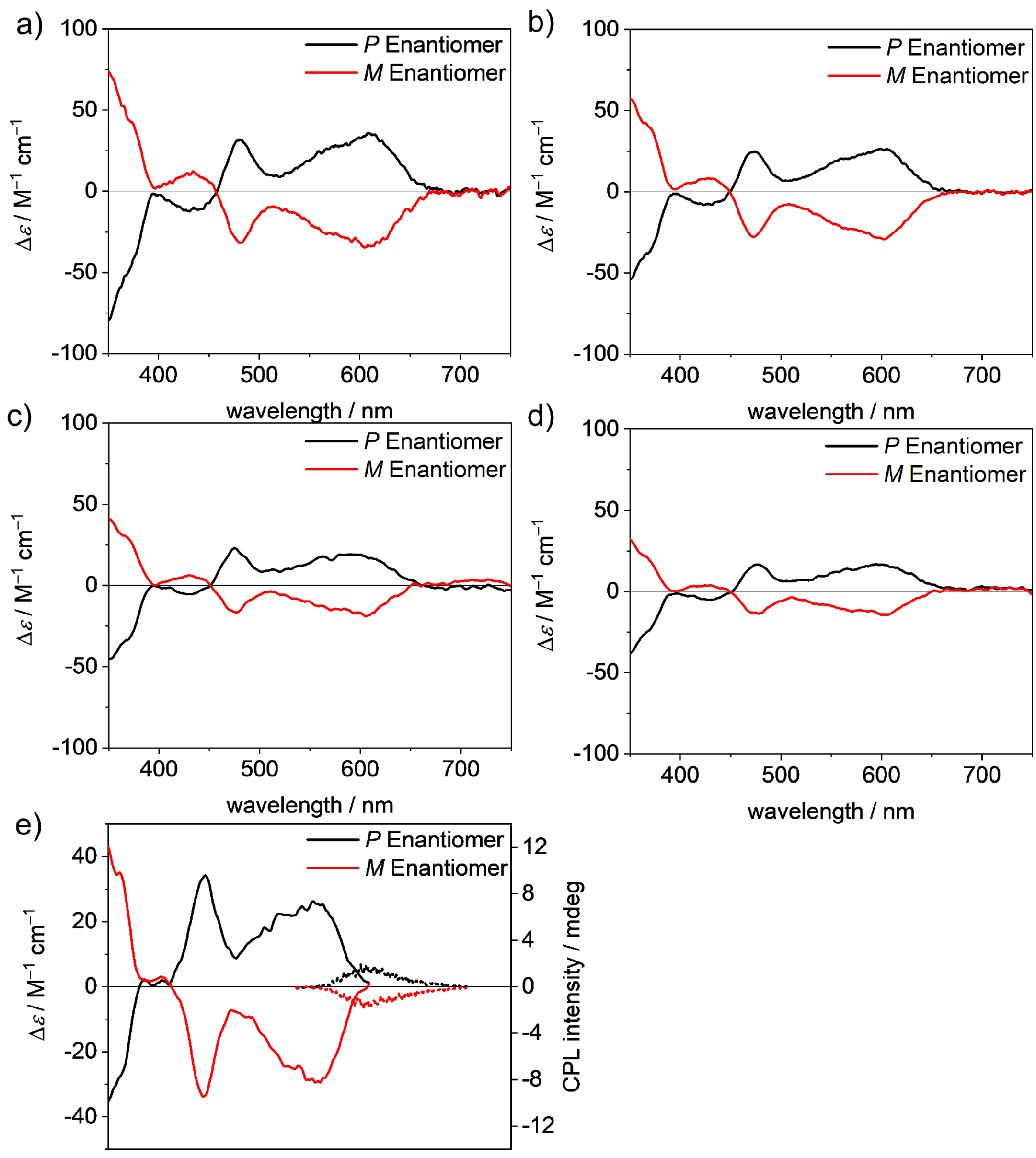

Figure S10 Circular dichroism (CD, c_0 = 1·10^{-5} M) spectra of *P*- (black) and *M*- (red) enantiomers of PBIs a) **2b**, b) **2c**, c) **2d**, d) **2e** and e) **3a** as well as circular polarized luminescence (CPL, c_0 = 2·10^{-6} M) spectra of **3a** in DCM at 293 K.

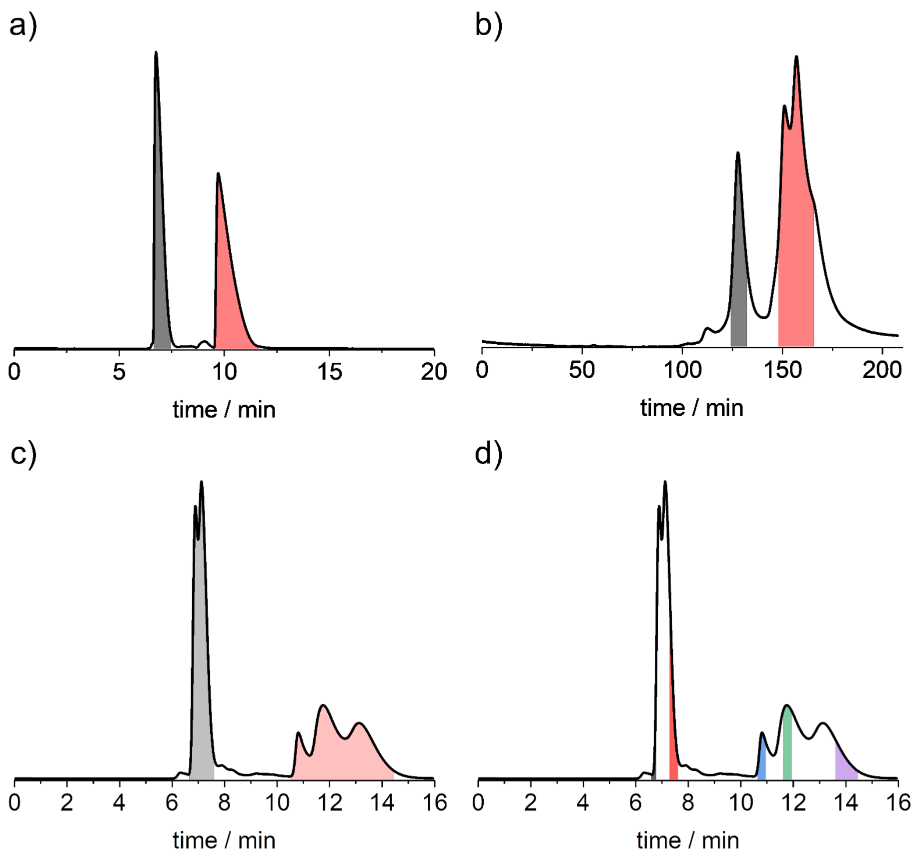

Figure S11 Elugrams of the HPLC separation on a chiral stationary phase of a) the successful separation of the atropoenantiomers of **4a**, and the non-successful separations of b) **6a**, as well as c) and d) for **5a** using mixtures of hexane and DCM as eluent. The collection times of the respective fractions are highlighted. Detection by UV/Vis analysis at 550 nm. According to CD spectral analysis, no separation of atropoenantiomers was achieved for **5a**. Thus, **5a** was for one run separated in c) two fractions and for another separated in d) five fractions. For each fraction a CD spectrum and a ^{1}H-NMR spectrum was measured, but no CD signals were obtained and the ^{1}H-NMR spectra all look identical to the spectrum shown in Figure S34. Similar results were obtained for **6a**.

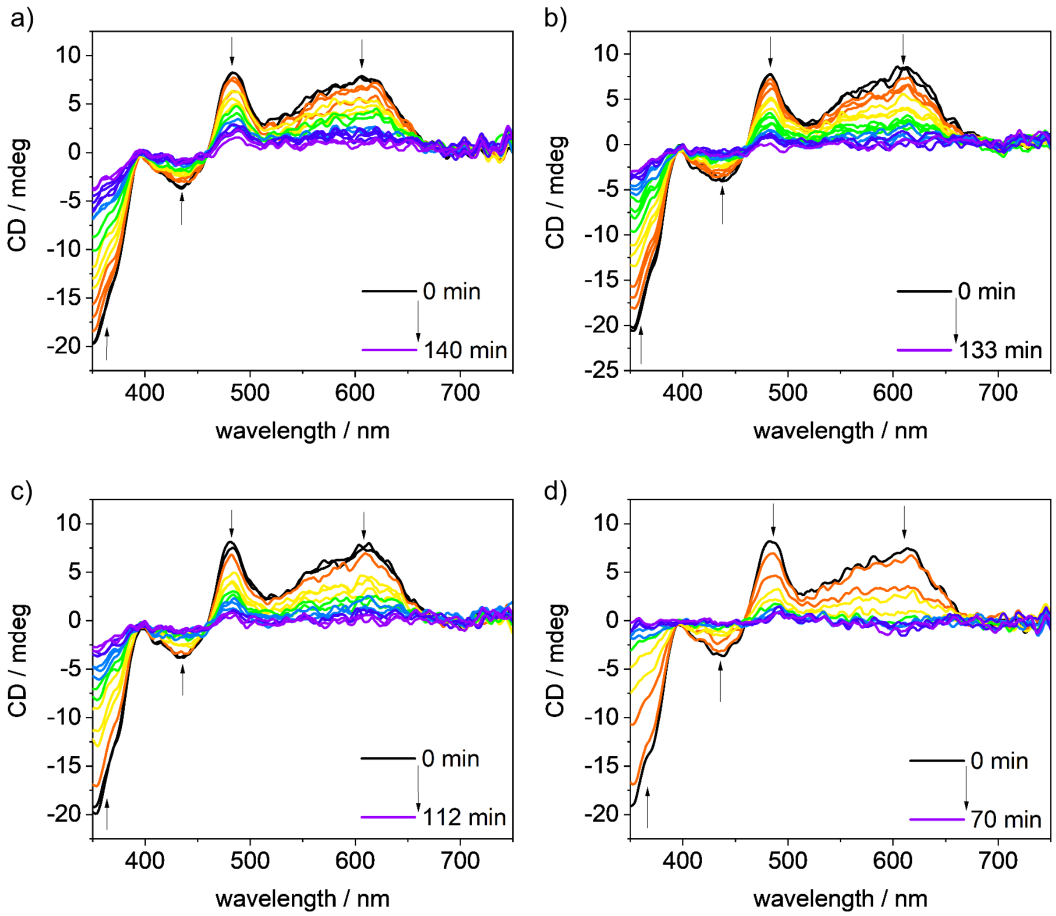

Figure S12 Time-dependent CD spectra of the *P*-enantiomer of **2a** in 1,1,2,2-tetrachloroethane at a) 383 K, b) 388 K, c) 393 K and d) 398 K.

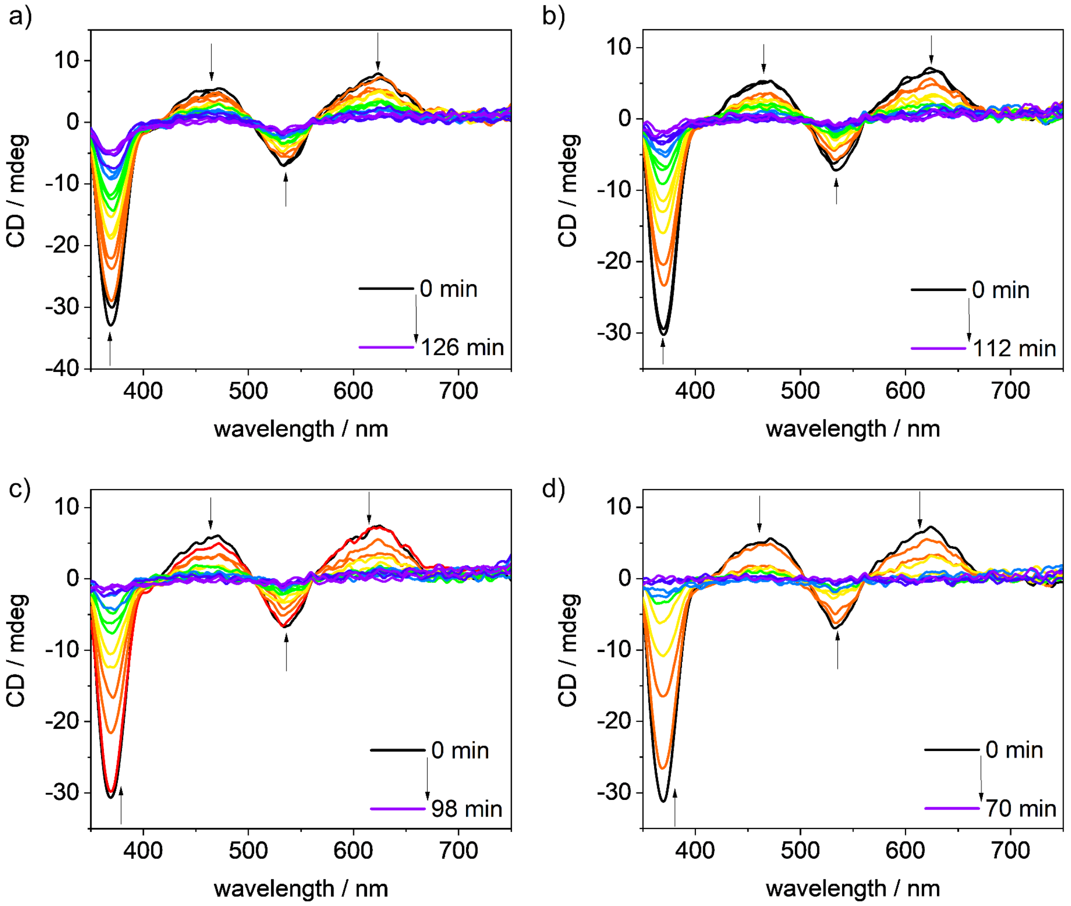

Figure S13 Time-dependent CD spectra of the *P*-enantiomer of **4a** in 1,1,2,2-tetrachloroethane at a) 383 K, b) 388 K, c) 393 K and d) 398 K.

Table S4 Kinetic data for the racemization process of **2a** and **4a** in 1,1,2,2-tetrachloroethane at four different temperatures obtained by CD spectroscopy.[S6]

**2a**				**4a**			
T **[K]**	***k*** **[10^{-5} s^{-1}]**	***t*$_{1/2}$ [min]**	**$\Delta G^{\ddagger}$ [kJ mol^{-1}]**	***T*** **[K]**	***k*** **[10^{-5} s^{-1}]**	***t*$_{1/2}$ [min]**	**$\Delta G^{\ddagger}$ [kJ mol^{-1}]**
383	19.7	59	119.6	383	27.1	43	118.6
388	33.1	35	119.8	388	38.5	30	119.3
393	43.1	27	120.2	393	59.1	20	119.2
398	87.2	13	119.5	398	82.8	14	119.6

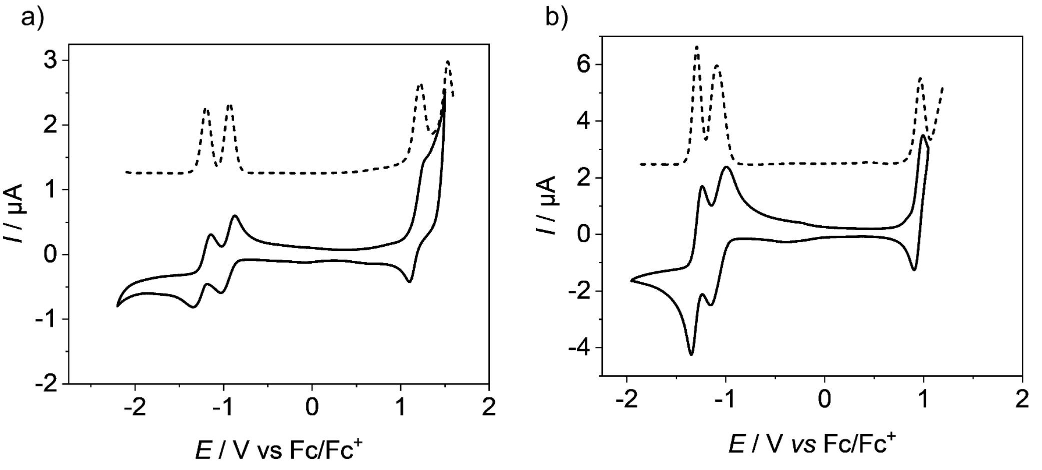

Figure S14 Cyclic voltammograms (solid lines) and square wave voltammograms (dashed lines) of PBIs a) **3a** and b) **4a**. Measurements were performed using DCM solutions ($c_0 \approx 2{\cdot}10^{-4}$ M) at 293 K, using tetrabutylammonium hexafluorophosphate (TBAHFP, 0.1 M) as electrolyte.

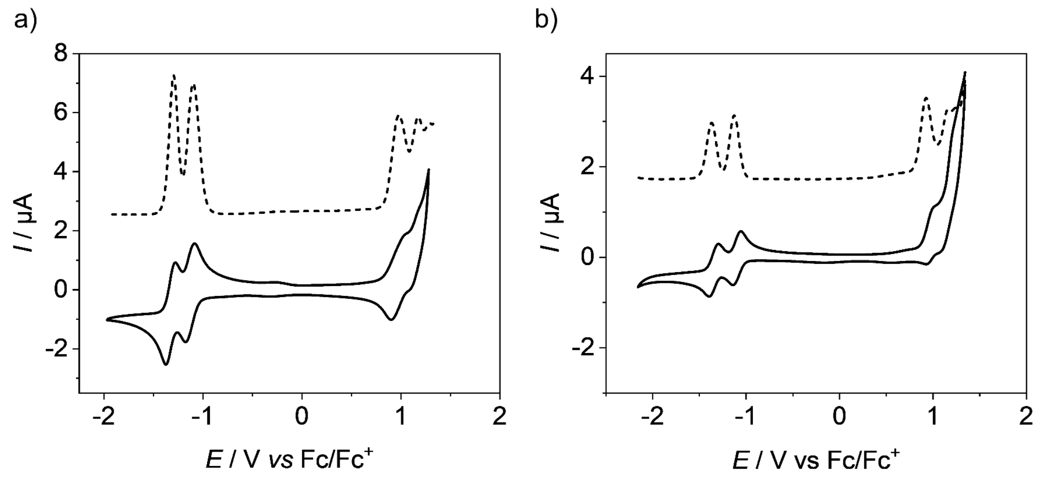

Figure S15 Cyclic voltammograms (solid lines) and square wave voltammograms (dashed lines) of PBI a) **5a** and b) **2b**. Measurements were performed using DCM solutions ($c_0 \approx 2{\cdot}10^{-4}$ M) at 293 K, using TBAHFP (0.1 M) as electrolyte.

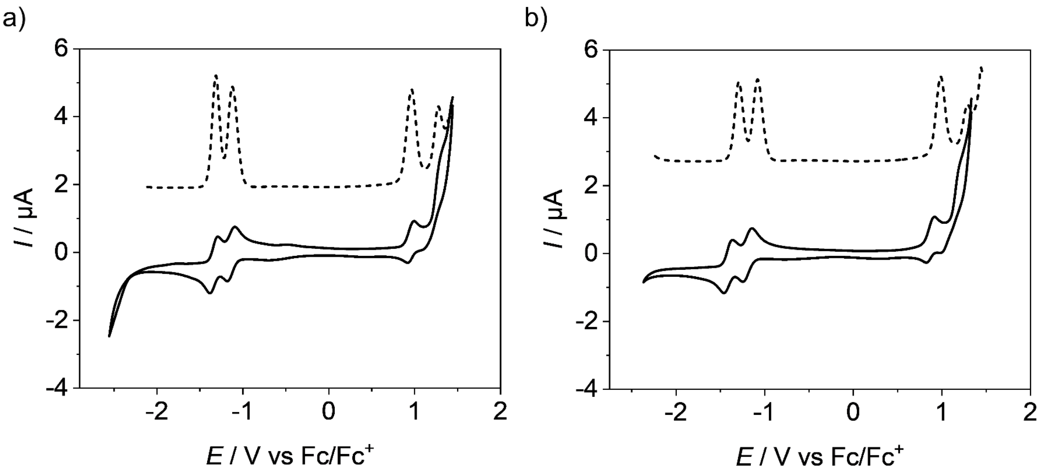

Figure S16 Cyclic voltammograms (solid lines) and square wave voltammograms (dashed lines) of a) **2c** and b) **2d**. Measurements were performed using DCM solutions ($c_0 \approx 2{\cdot}10^{-4}$ M) at 293 K, using TBAHFP (0.1 M) as electrolyte.

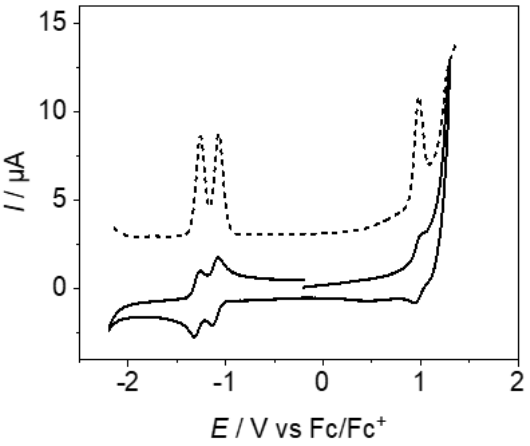

Figure S17 Cyclic voltammogram (solid lines) and square wave voltammogram (dashed lines) of **2e**. Measurements were performed using DCM solutions ($c_0 \approx 2{\cdot}10^{-4}$ M) at 293 K, using TBAHFP (0.1 M) as electrolyte.

Table S5 Summary of the redox properties of **2a** - **2e**. Half-wave potentials were determined by cyclic or square wave voltammetry measured in DCM (0.1 M TBAHFP) vs. Fc/Fc^+ at 293 K.

PBI	E_{red1} [V]	E_{red2} [V]	E_{ox1} [V]	E_{ox2} [V]	E_{HOMO}[a] [eV]	E_{LUMO}[a] [eV]	E_{gap} [eV]
**2a**	–1.05	–1.21	0.97	-[g]	–6.12	–4.10	2.02
**2b**	–1.02	–1.25	1.01	1.19	–6.16	–4.13	2.03
**2c**	–1.12	–1.31	0.96	1.28	–6.11	–4.03	2.08
**2d**	–1.08	–1.29	0.99	1.29	–6.14	–4.07	2.07
**2e**	–1.07	–1.26	0.97	-[b]	–6.12	–4.08	2.04
**7**	–1.00	–1.22	1.30	-[b]	–6.45	–4.15	2.30
**8**	–1.10	–1.29	0.90	1.21	–6.05	–4.05	2.00
Pyrene	-[b]	-[b]	0.63	-[b]	–5.78	-	-

[a] Calculated according to literature known procedure using the experimentally determined redox potentials (E_{HOMO}= –[E_{ox1} +5.15 eV] and E_{LUMO} = –[E_{red1} +5.15 eV]) and the energy level of Fc/Fc^+ with respect to the vacuum level (–5.15 eV). [b] Not observed.

Table S6 Calculated UV/Vis/NIR transitions for compound **2** using TD-DFT (B3LYP/def2-SVP).

Transition	% Character	Wavelength / nm	Oscillator strength
188 → 189	100	669.44	0.2596
187 → 189	100	485.49	0.0420
186 → 189<br>188 → 192	97.3<br>2.7	472.05	0.2050
184 → 189	100	412.04	0.0087

Table S7 Calculated UV/Vis/NIR transitions for compound **3** using TD-DFT (B3LYP/def2-SVP).

Transition	% Character	Wavelength / nm	Oscillator strength
252 → 253	100	792.38	0.1358
251 → 253	100	532.93	0.0272
248 → 253<br>250 → 253<br>252 → 256	26.4<br>68.9<br>4.7	471.17	0.1506
248 → 253<br>250 → 253<br>252 → 256	72.6<br>25.2<br>2.2	467.75	0.0622
239 → 253<br>246 → 253<br>252 → 254<br>252 → 255	10.3<br>42.3<br>26.4<br>21.0	407.63	0.0033
239 → 253<br>246 → 253<br>252 → 254<br>252 → 255	36.8<br>15.3<br>24.7<br>23.2	399.83	0.0194
237 → 253<br>238 → 253<br>244 → 253<br>247 → 253	4.0<br>5.6<br>8.5<br>81.9	397.19	0.0154

Table S8 Calculated UV/Vis/NIR transitions for compound **4** using TD-DFT (B3LYP/def2-SVP).

Transition	% Character	Wavelength / nm	Oscillator strength
240 → 241	100	760.46	0.1012
239 → 241	100	624.61	0.0573
238 → 241	100	623.55	0.0431
236 → 241	100	510.93	0.1211
235 → 241	100	466.05	0.2160
234 → 241<br>240 → 243	97.7<br>2.3	436.71	0.0248
230 → 241<br>234 → 241<br>240 → 243<br>240 → 247	3.0<br>2.1<br>92.6<br>2.3	400.05	0.0525

Table S9 Calculated UV/Vis/NIR transitions for compound **5** using TD-DFT (B3LYP/def2-SVP).

Transition	% Character	Wavelength / nm	Oscillator strength
236 → 241<br>240 → 241	3.6<br>96.4	693.53	0.1344
239 → 241	100	658.56	0.0699
237 → 241	100	599.36	0.0292
236 → 241<br>240 → 241	96.5<br>3.5	499.27	0.1542
234 → 241	100	420.41	0.0031
226 → 241<br>233 → 241<br>240 → 245	2.3<br>93.8<br>3.9	402.74	0.0088
232 → 241<br>240 → 243	48.2<br>51.8	396.86	0.0066

Table S10 Calculated UV/Vis/NIR transitions for compound **6** using TD-DFT (B3LYP/def2-SVP).

Transition	% Character	Wavelength / nm	Oscillator strength
316 → 317	100	907.21	0.0101
315 → 317	100	885.97	0.1173
313 → 317	100	743.48	0.0491
312 → 317	100	513.35	0.0619
311 → 317<br>316 → 318	20.4<br>79.6	466.17	0.0404
311 → 317<br>316 → 318	79.4<br>20.6	453.49	0.2214

4. NMR spectra

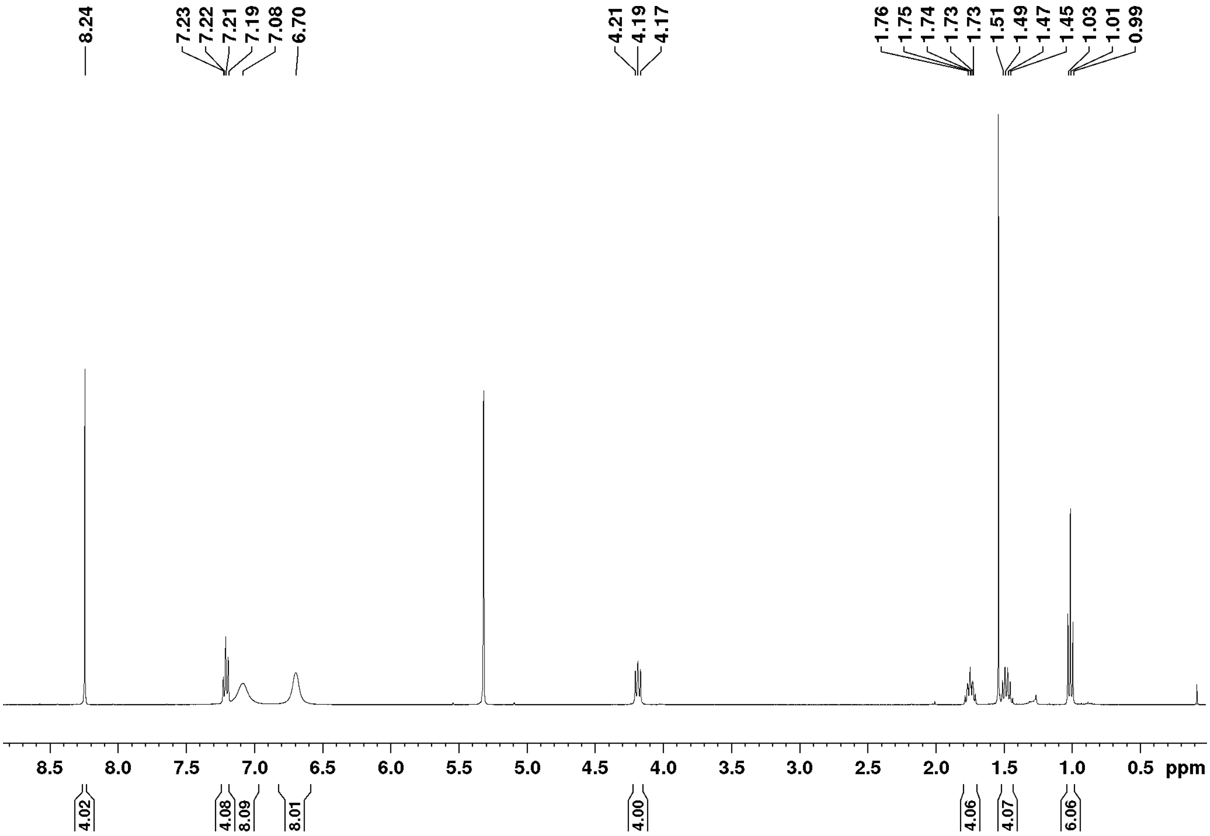

Figure S18 ^{1}H-NMR spectrum of **2a** in CD_2Cl_2 recorded at 295 K.

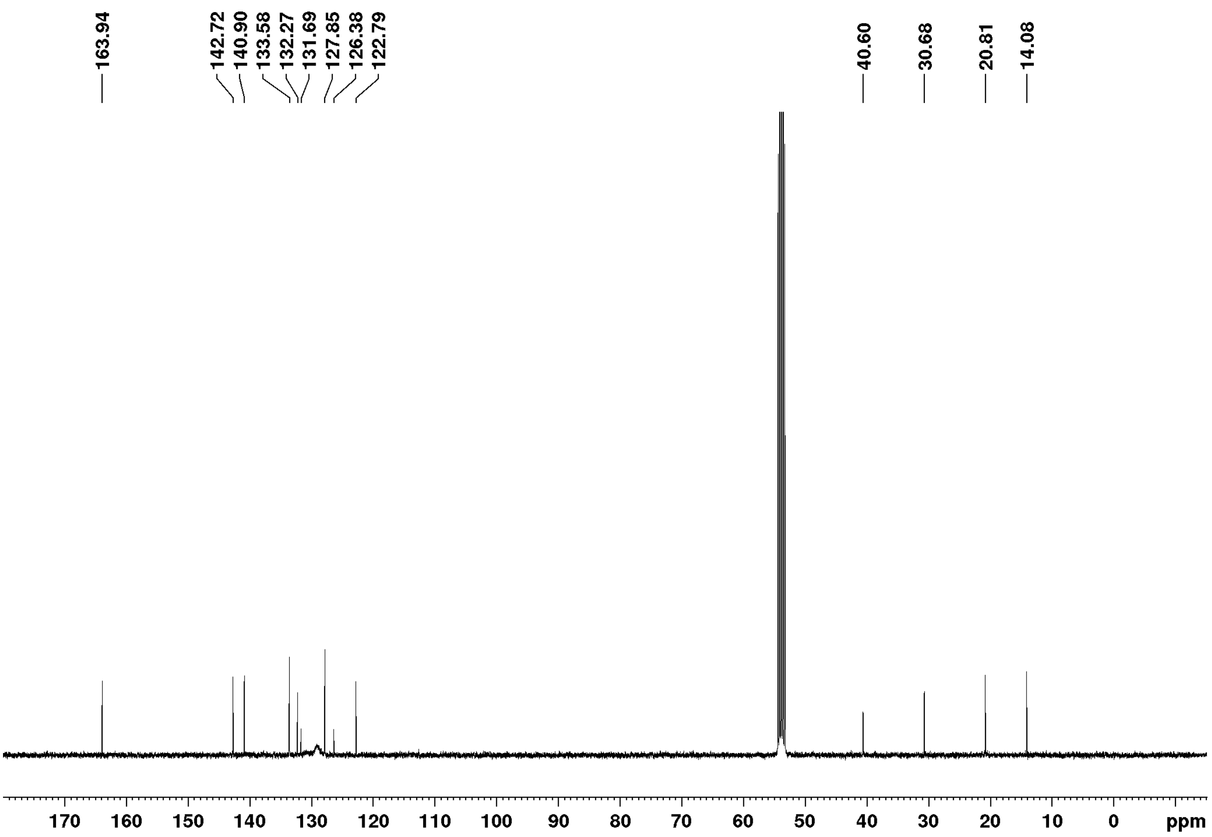

Figure S19 ^{13}C-NMR spectrum of **2a** in CD_2Cl_2 recorded at 295 K.

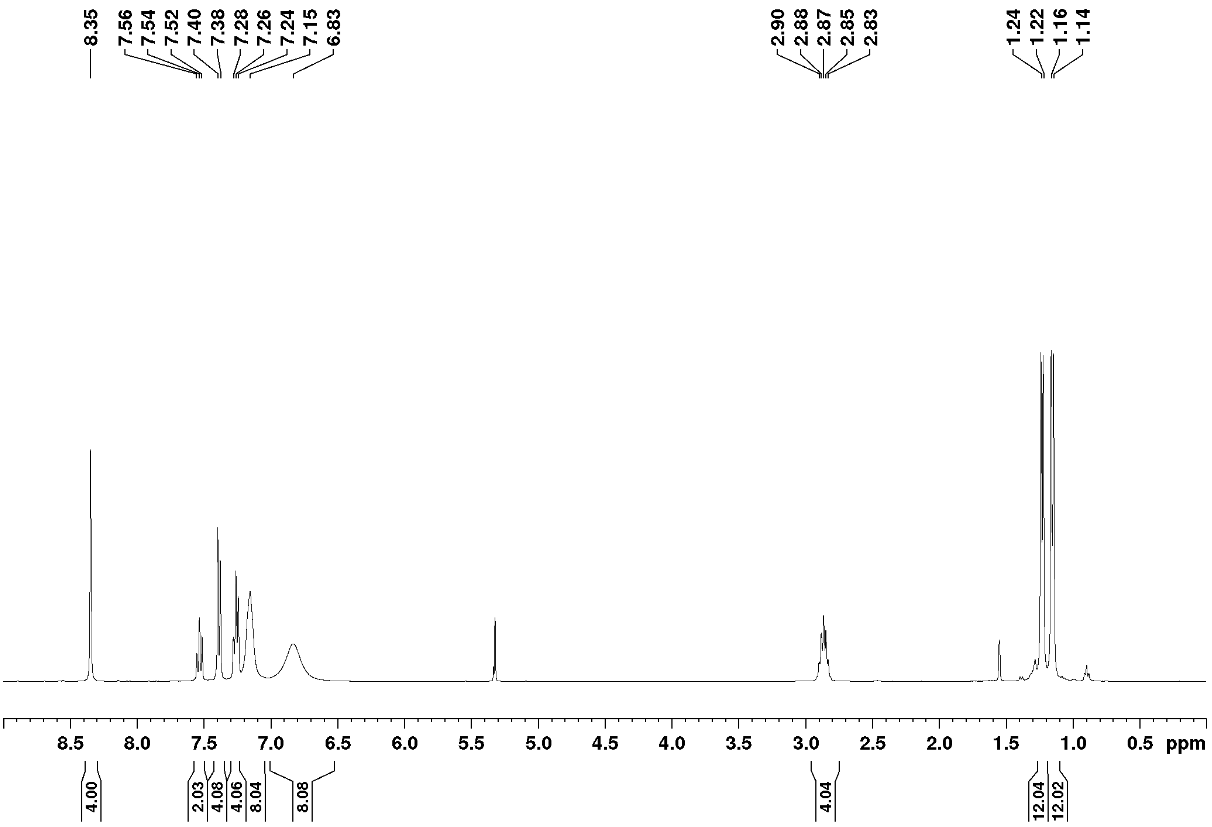

Figure S20 ^{1}H-NMR spectrum of **2b** in CD_2Cl_2 recorded at 295 K.

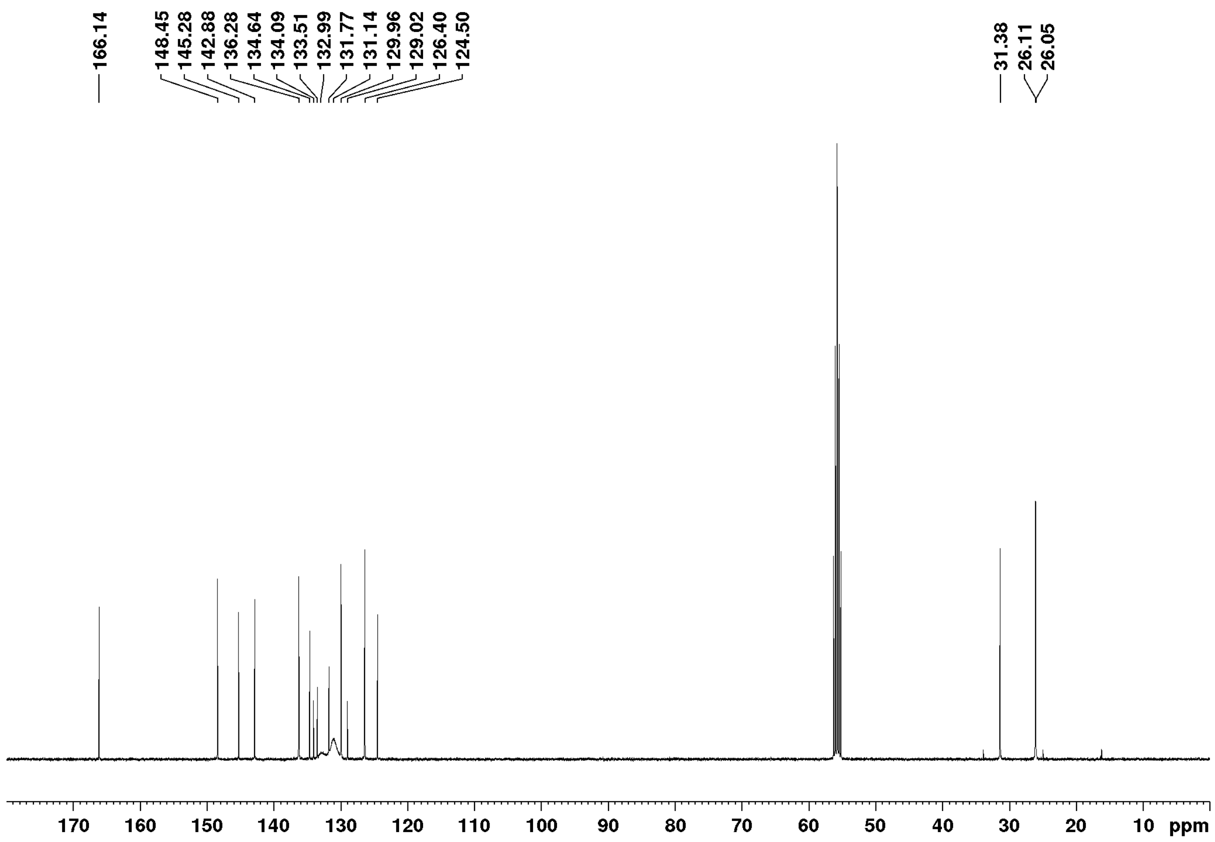

Figure S21 ^{13}C-NMR spectrum of **2b** in CD_2Cl_2 recorded at 295 K.

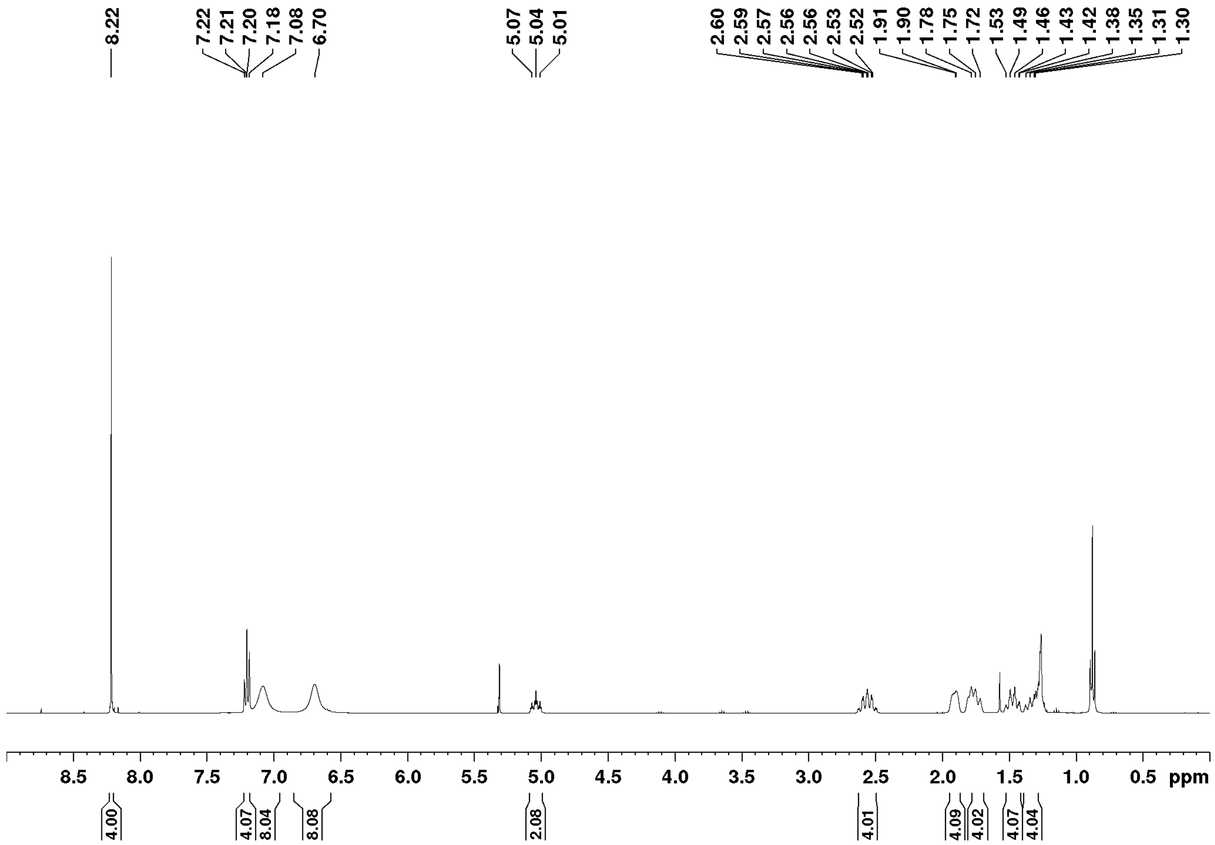

Figure S22 ^{1}H-NMR spectrum of **2c** in CD_2Cl_2 recorded at 295 K.

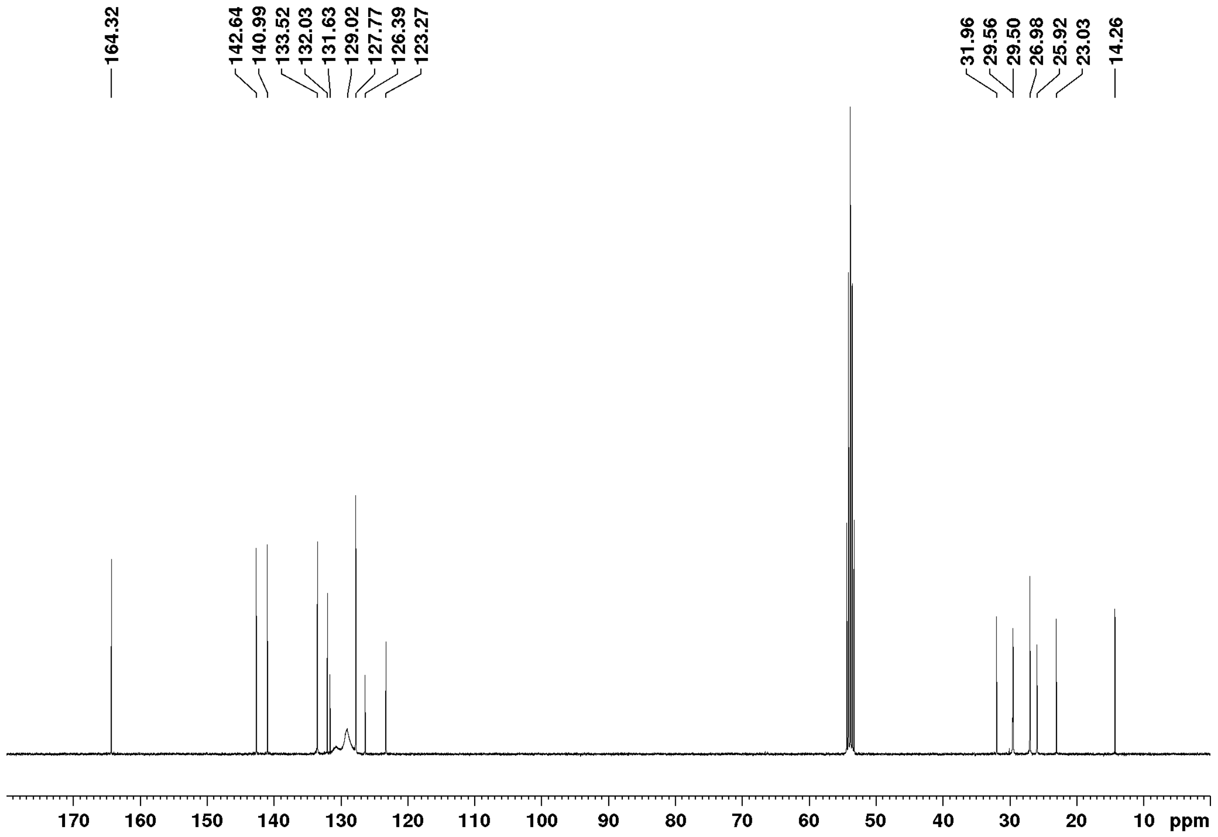

Figure S23 ^{13}C-NMR spectrum of **2c** in CD_2Cl_2 recorded at 295 K.

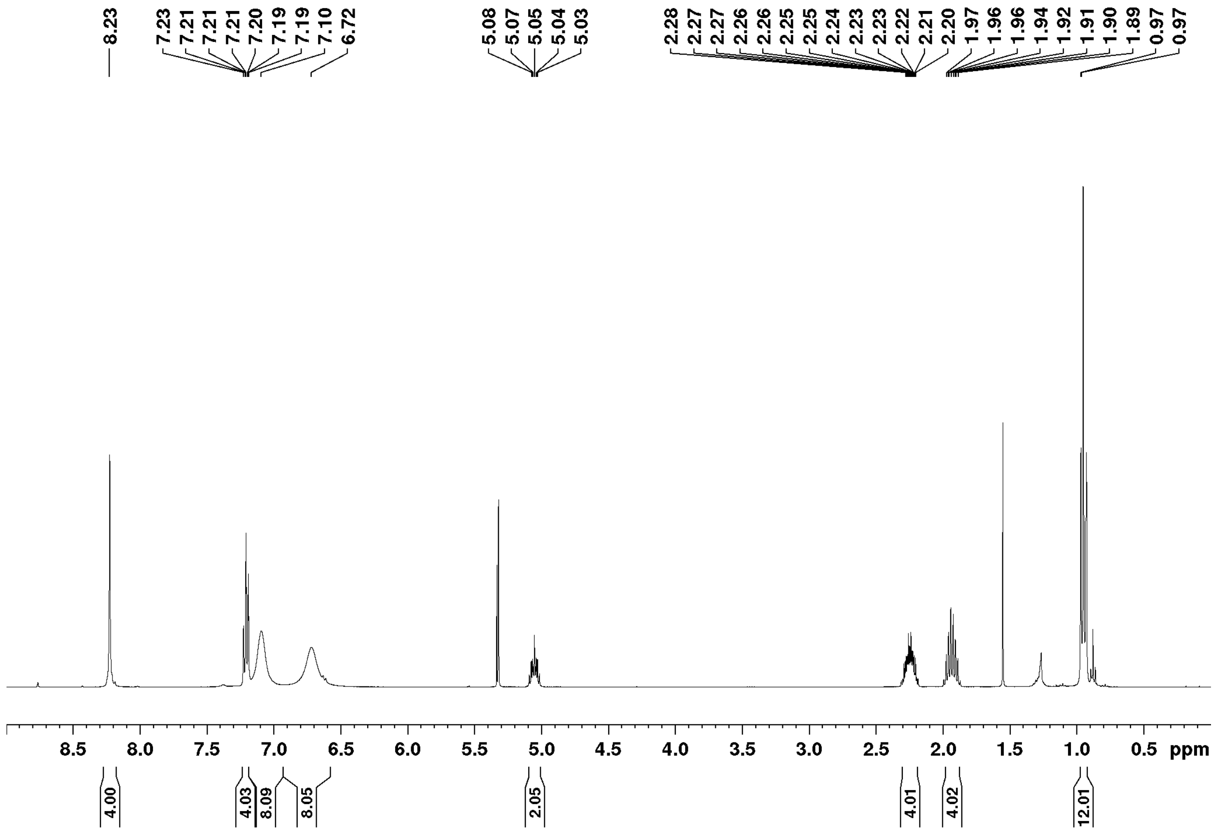

Figure S24 ^{1}H-NMR spectrum of **2d** in CD_2Cl_2 recorded at 295 K.

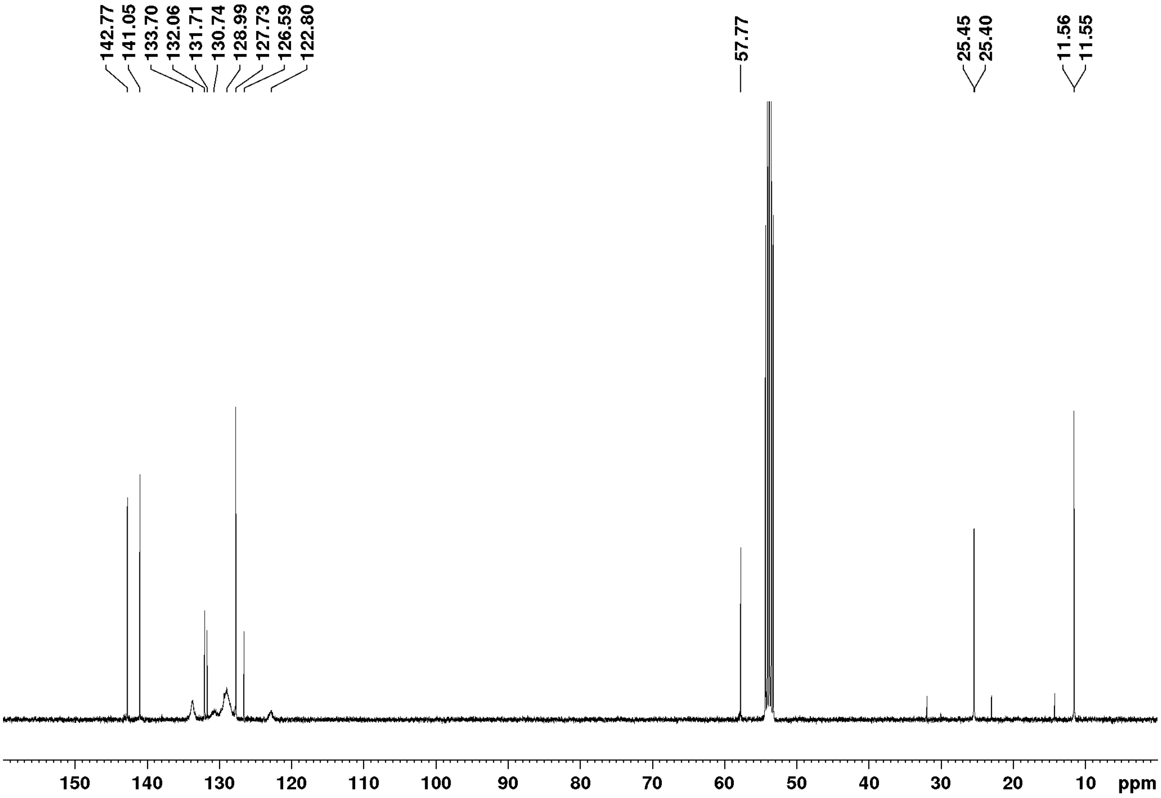

Figure S25 ^{13}C-NMR spectrum of **2d** in CD_2Cl_2 recorded at 295 K.

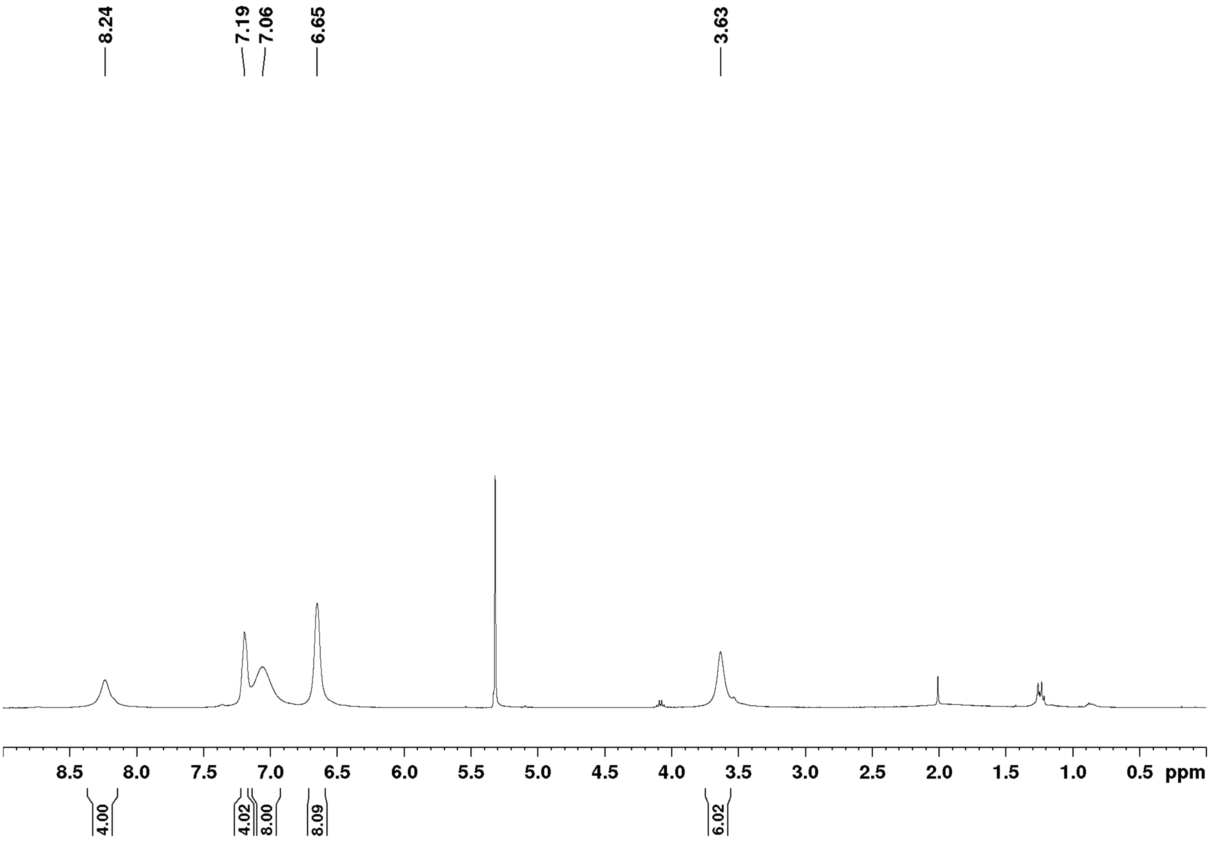

Figure S26 ^{1}H-NMR spectrum of **2e** in CD_2Cl_2 recorded at 295 K.

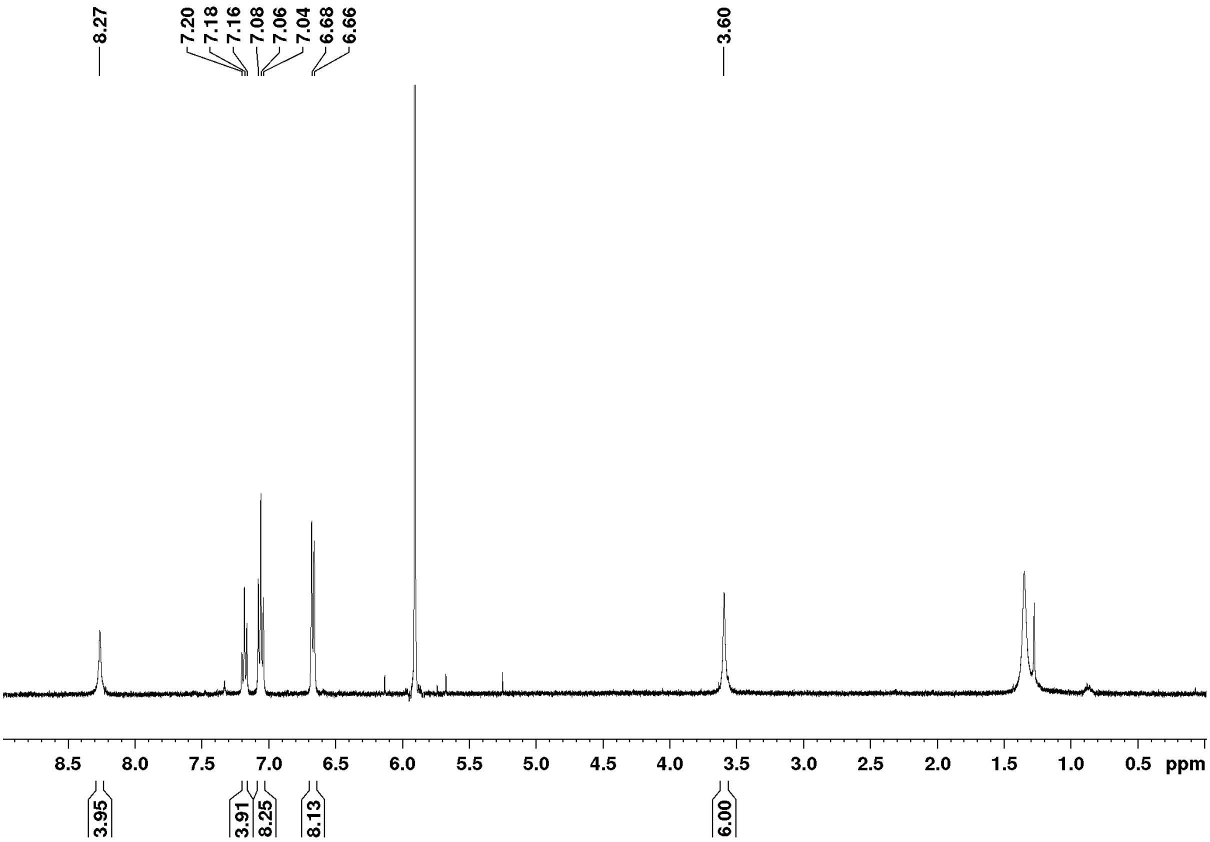

Figure S27 ^{1}H-NMR spectrum of **2e** in $C_2D_2Cl_4$ recorded at 390 K.

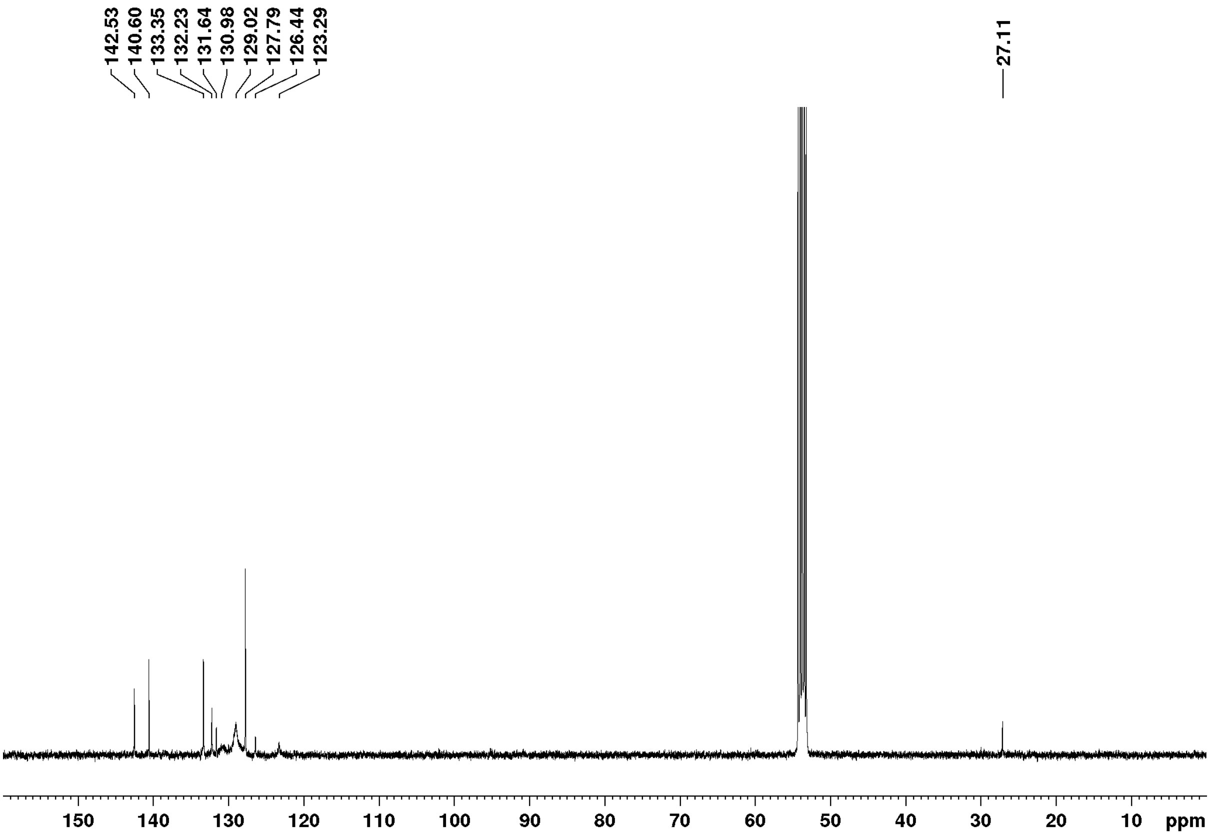

Figure S28 ^{13}C-NMR spectrum of **2e** in CD_2Cl_2 recorded at 295 K.

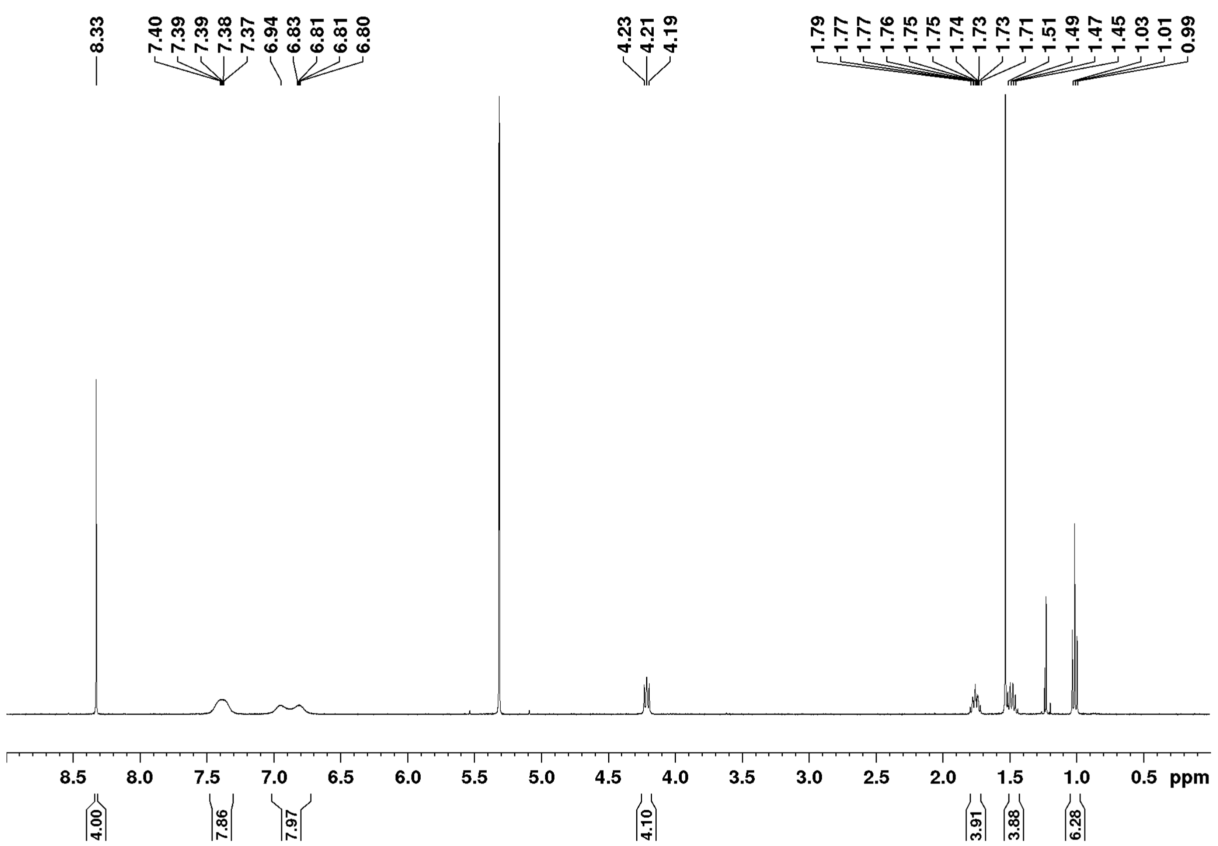

Figure S29 ^{1}H-NMR spectrum of **3a** in CD_2Cl_2 recorded at 295 K.

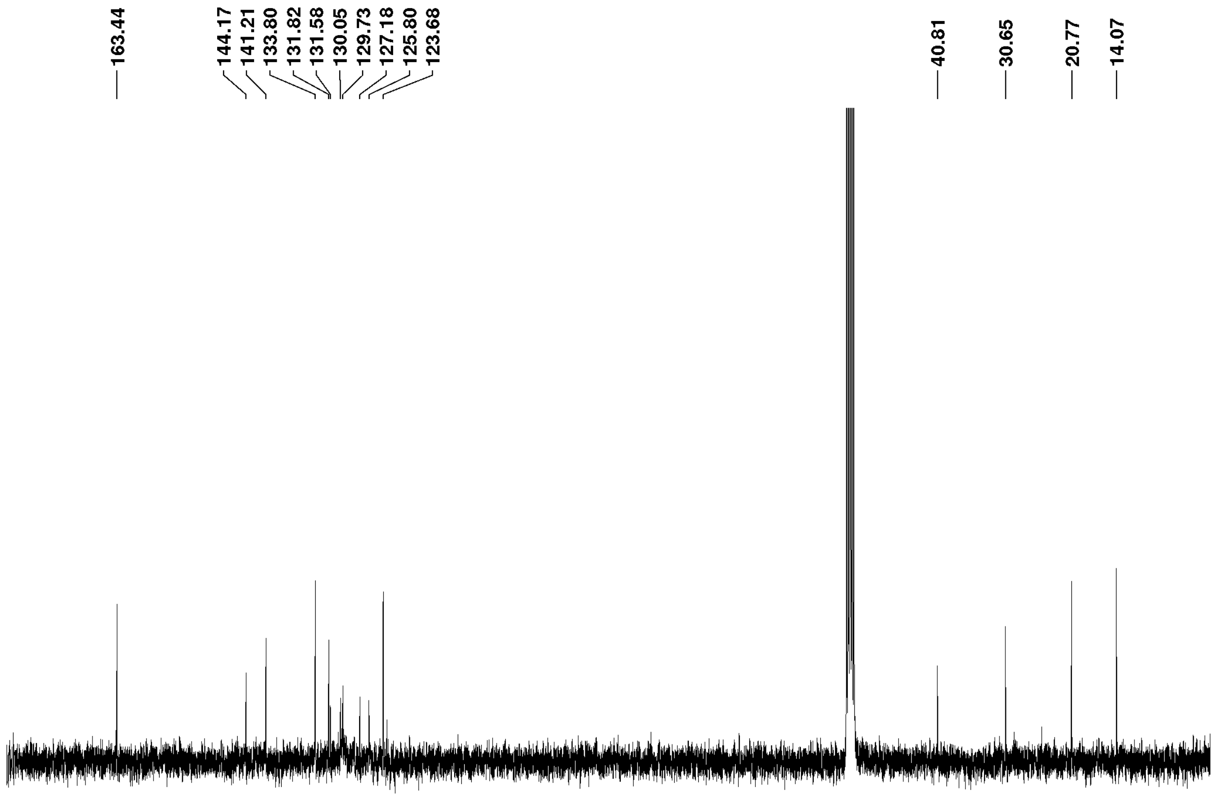

Figure S30 ^{13}C-NMR spectrum of **3a** in CD_2Cl_2 recorded at 295 K.

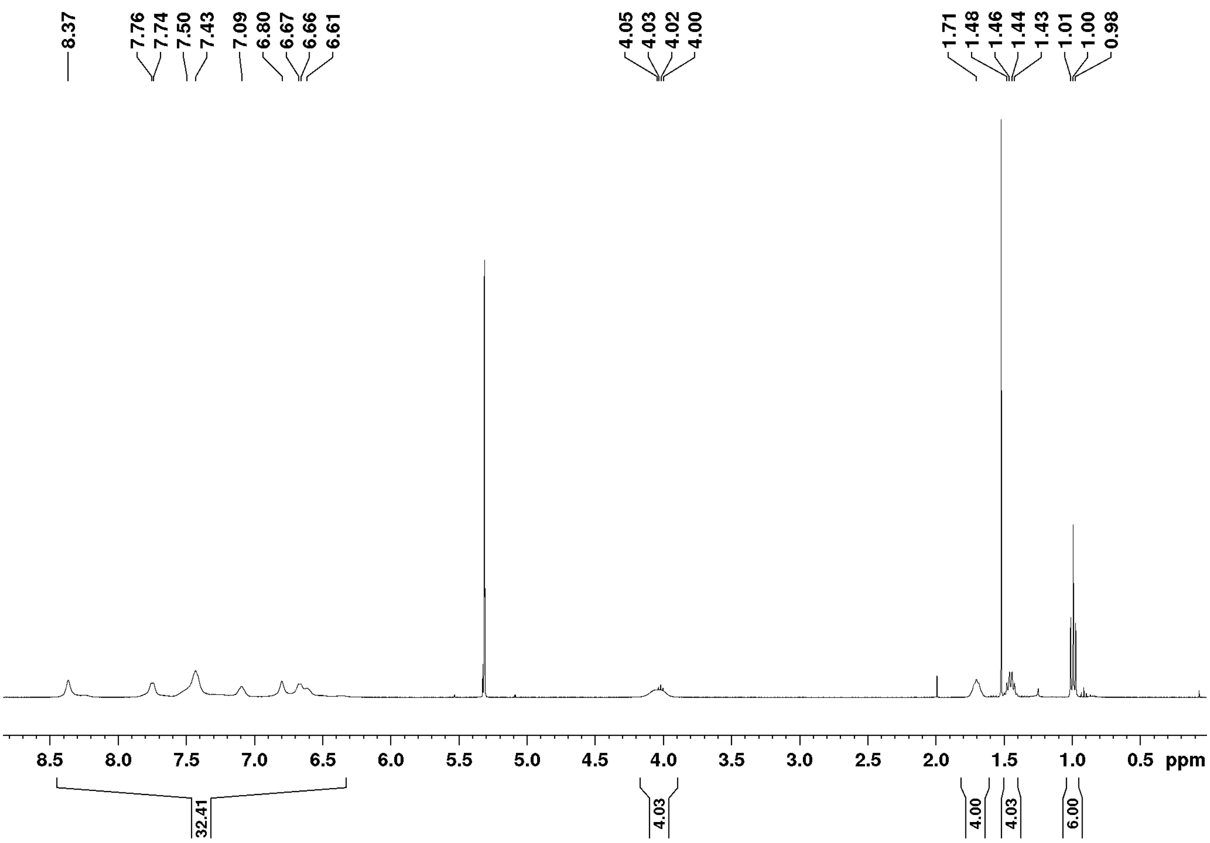

Figure S31 ^{1}H-NMR spectrum of **4a** in CD_2Cl_2 recorded at 295 K.

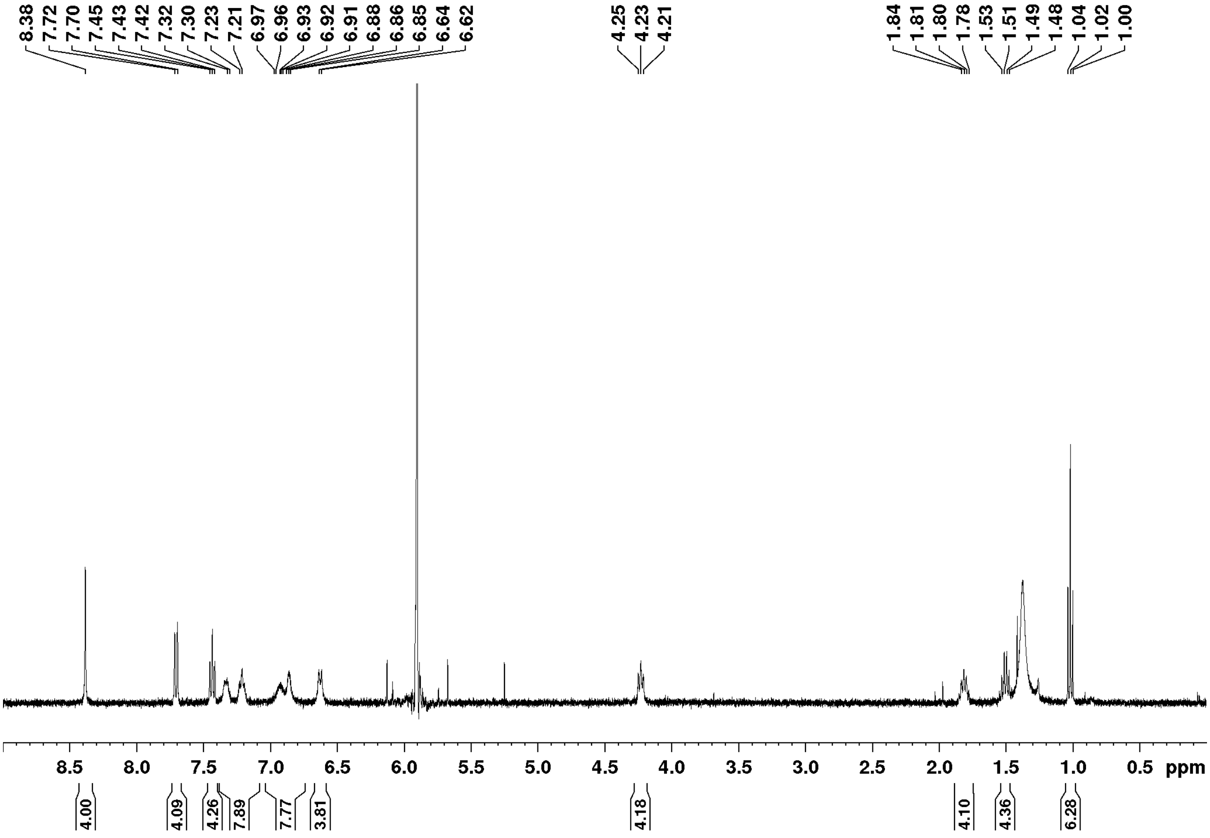

Figure S32 ^{1}H-NMR spectrum of **4a** in $C_2D_2Cl_4$ recorded at 390 K.

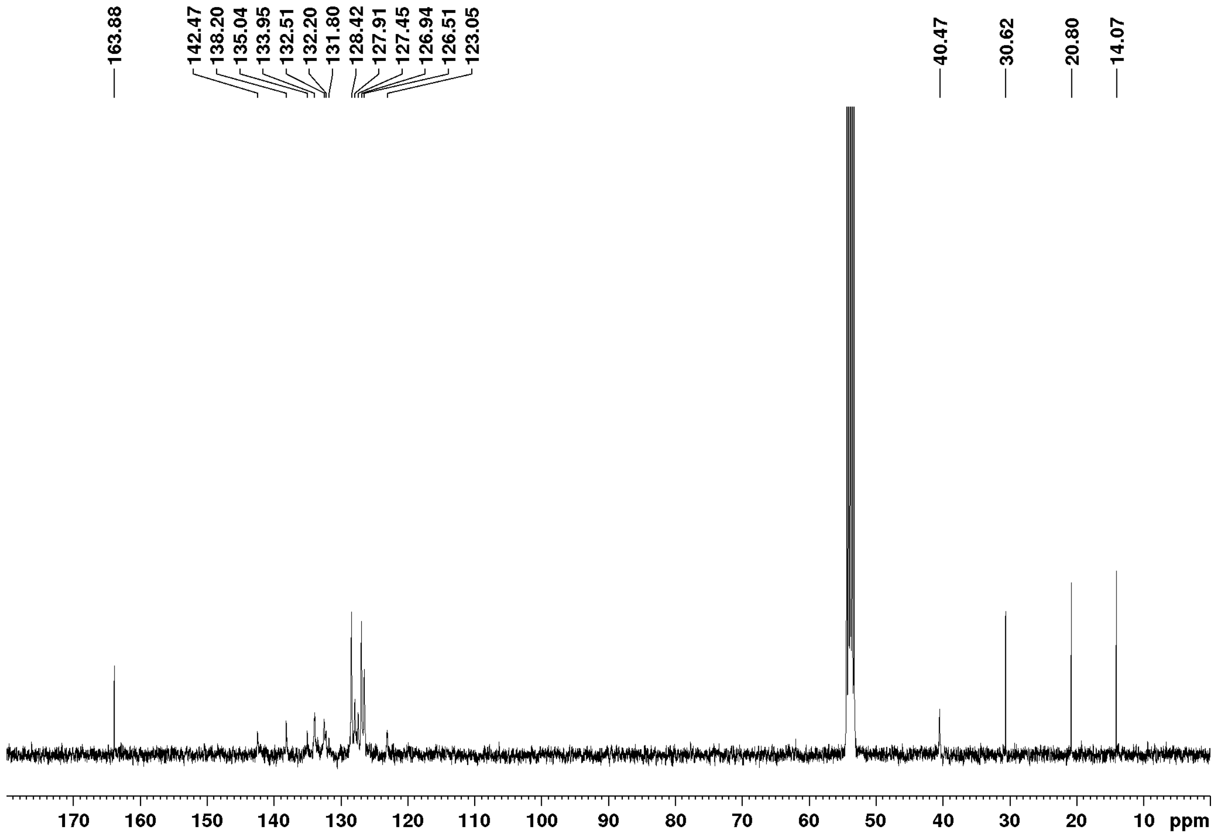

Figure S33 ^{13}C-NMR spectrum of **4a** in CD_2Cl_2 recorded at 295 K.

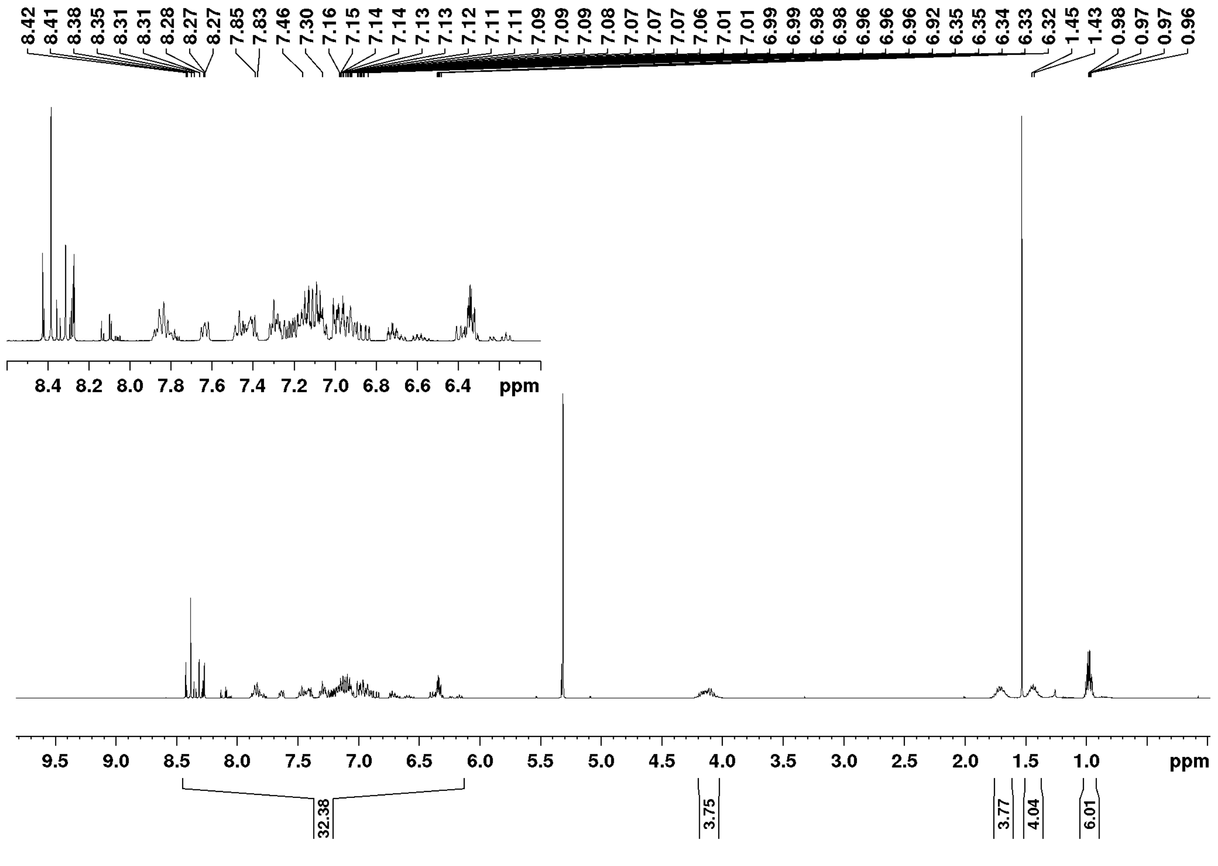

Figure S34 ^{1}H-NMR spectrum of **5a** in CD_2Cl_2 recorded at 295 K.

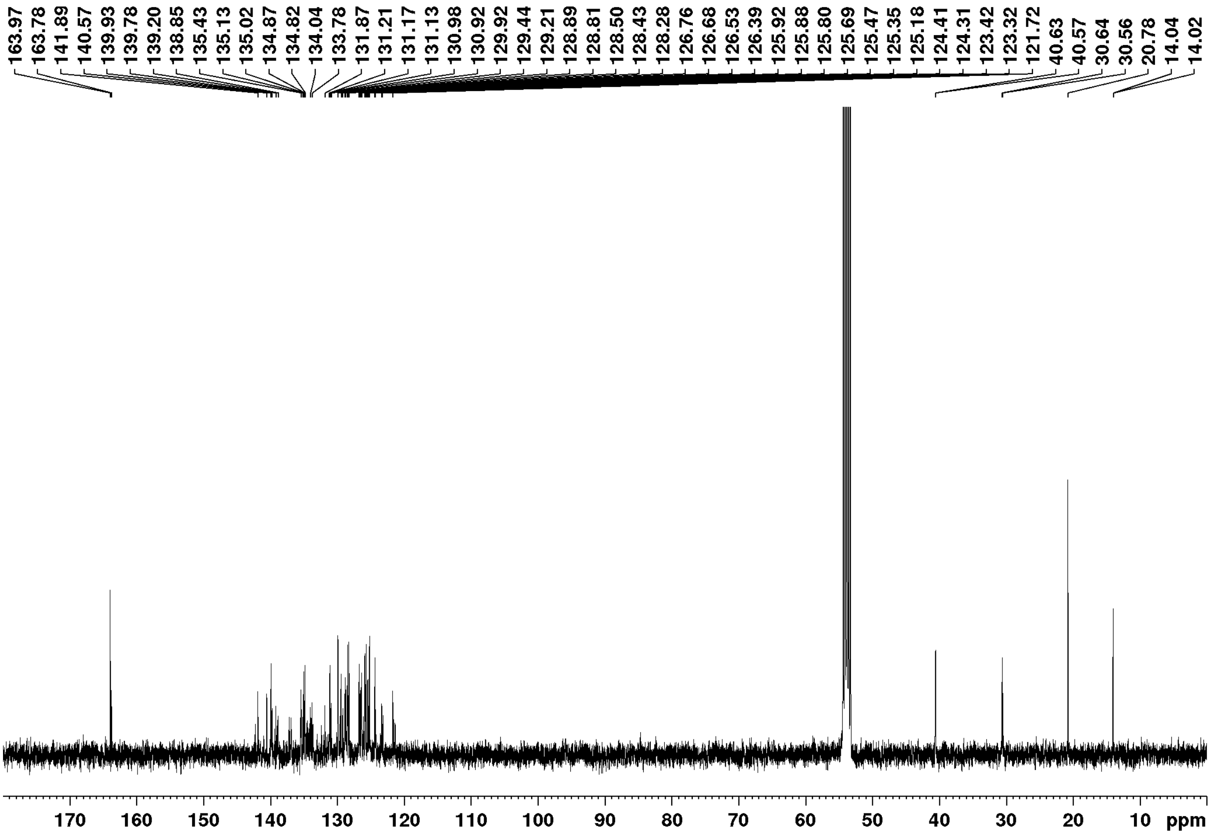

Figure S35 ^{13}C-NMR spectrum of **5a** in CD_2Cl_2 recorded at 295 K.

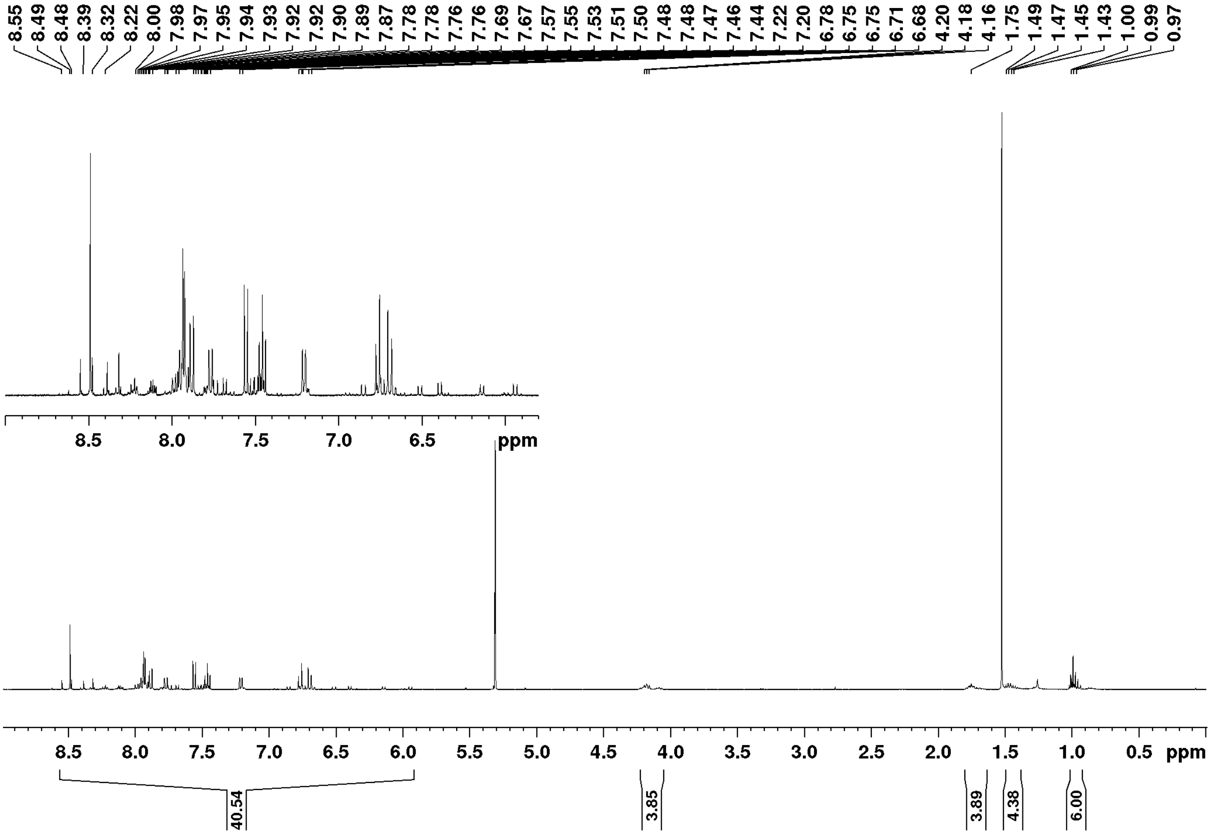

Figure S36 ^{1}H-NMR spectrum of **6a** in CD_2Cl_2 recorded at 295 K.

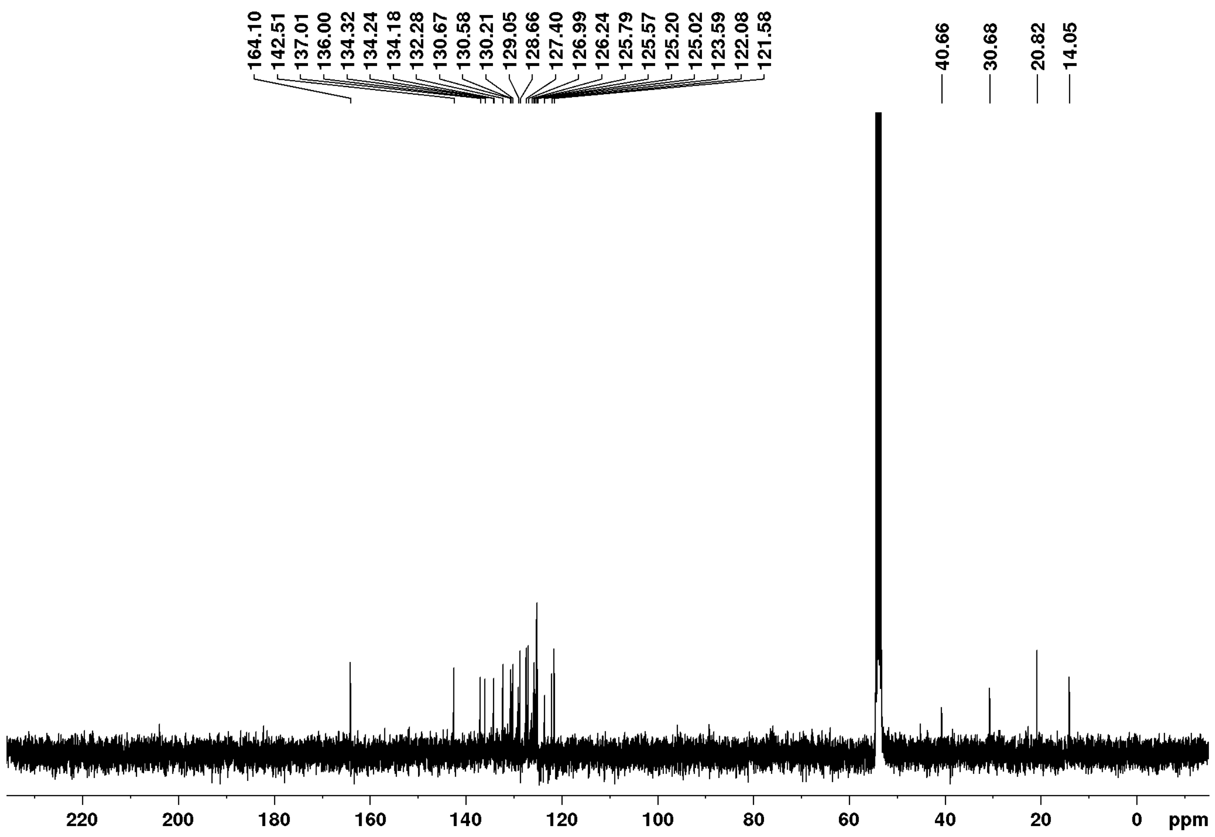

Figure S37 ^{13}C-NMR spectrum of **6a** in CD_2Cl_2 recorded at 295 K.

5. Supporting References

[S1] a) F. Würthner, A. Sautter, J. Schilling, *J. Org. Chem.* **2002**, *67*, 3037-3044; b) B. Pagoaga, L. Giraudet, N. Hoffmann, *Eur. J. Org. Chem.* **2014**, 5178-5195; c) S. Mathew, M. R. Johnston, *Chem. Eur. J.* **2009**, *15*, 248-253; d) K. Mahata, P. D. Frischmann, F. Würthner, *J. Am. Chem. Soc.* **2013**, *135*, 15656-15661.

[S2] G. M. Sheldrick, *Acta Crystallogr. A* **2008**, 64, 112-122.

[S3] M. J. Frisch, G. W. Trucks, H. B. Schlegel, G. E. Scuseria, M. A. Robb, J. R. Cheeseman, G. Scalmani, V. Barone, G. A. Petersson, H. Nakatsuji, X. Li, M. Caricato, A. V. Marenich, J. Bloino, B. G. Janesko, R. Gomperts, B. Mennucci, H. P. Hratchian, J. V. Ortiz, A. F. Izmaylov, J. L. Sonnenberg, Williams, F. Ding, F. Lipparini, F. Egidi, J. Goings, B. Peng, A. Petrone, T. Henderson, D. Ranasinghe, V. G. Zakrzewski, J. Gao, N. Rega, G. Zheng, W. Liang, M. Hada, M. Ehara, K. Toyota, R. Fukuda, J. Hasegawa, M. Ishida, T. Nakajima, Y. Honda, O. Kitao, H. Nakai, T. Vreven, K. Throssell, J. A. Montgomery Jr., J. E. Peralta, F. Ogliaro, M. J. Bearpark, J. J. Heyd, E. N. Brothers, K. N. Kudin, V. N. Staroverov, T. A. Keith, R. Kobayashi, J. Normand, K. Raghavachari, A. P. Rendell, J. C. Burant, S. S. Iyengar, J. Tomasi, M. Cossi, J. M. Millam, M. Klene, C. Adamo, R. Cammi, J. W. Ochterski, R. L. Martin, K. Morokuma, O. Farkas, J. B. Foresman, D. J. Fox, Wallingford, CT, **2016**.

[S4] J. Merz, J. Fink, A. Friedrich, I. Krummenacher, H. H. Al Mamari, S. Lorenzen, M. Hähnel, A. Eichhorn, M. Moos, M. Holzapfel, H. Braunschweig, C. Lambert, A. Steffen, L. Ji, T. B. Marder, *Chem. Eur. J.* **2017**, *23*, 13164-13180.

[S5] R. Sens, K. H. Drexhage, *J. Lumin.* **1981**, *24*, 709-712.

[S6] P. Osswald, F. Würthner, *J. Am. Chem. Soc.* **2007**, *129*, 14319-14326.
